# EPS Glycoconjugate Profiles Shift as Adaptive Response in Anaerobic Microbial Granulation at High Salinity

**DOI:** 10.3389/fmicb.2018.01423

**Published:** 2018-07-02

**Authors:** Maria C. Gagliano, Thomas R. Neu, Ute Kuhlicke, Dainis Sudmalis, Hardy Temmink, Caroline M. Plugge

**Affiliations:** ^1^Laboratory of Microbiology, Wageningen University & Research, Wageningen, Netherlands; ^2^Helmholtz Centre for Environmental Research, Magdeburg, Germany; ^3^Sub-department of Environmental Technology, Wageningen University & Research, Wageningen, Netherlands

**Keywords:** granular sludge, EPS, *Methanosaeta*, high salinity, anaerobic digestion, lectin staining, biofilm

## Abstract

Anaerobic granulation at elevated salinities has been discussed in several analytical and engineering based studies. They report either enhanced or decreased efficiencies in relation to different Na^+^ levels. To evaluate this discrepancy, we focused on the microbial and structural dynamics of granules formed in two upflow anaerobic sludge blanket (UASB) reactors treating synthetic wastewater at low (5 g/L Na^+^) and high (20 g/L Na^+^) salinity conditions. Granules were successfully formed in both conditions, but at high salinity, the start-up inoculum quickly formed larger granules having a thicker gel layer in comparison to granules developed at low salinity. Granules retained high concentrations of sodium without any negative effect on biomass activity and structure. 16S rRNA gene analysis and Fluorescence *in Situ* Hybridization (FISH) identified the acetotrophic *Methanosaeta harundinacea* as the dominant microorganism at both salinities. Fluorescence lectin bar coding (FLBC) screening highlighted a significant shift in the glycoconjugate pattern between granules grown at 5 and 20 g/L of Na^+^, and the presence of different extracellular domains. The excretion of a Mannose-rich cloud-like glycoconjugate matrix, which seems to form a protective layer for some methanogenic cells clusters, was found to be the main distinctive feature of the microbial community grown at high salinity conditions.

## Introduction

The upflow anaerobic sludge blanket (UASB) reactor, designed in 1970s by Lettinga et al. (1980), is commonly applied to treat high-strength industrial wastewaters (Van Lier et al., 2015). In such reactors microbial biomass spontaneously aggregates into granules with a characteristic structure (Sekiguchi et al., 1999), and this enables high organic removal and methane production rates (Seghezzo et al., 1998; Hulshoff Pol et al., 2004).

In granules, microorganisms produce a significant amount of extracellular polymeric substances (EPS) to form a hydrogel matrix, similarly as in biofilms (Pronk et al., 2017). EPS perform an important role in defining the physical properties of microbial aggregates, where the excreted structural glycoconjugates can influence the complex spatial architecture (Seviour et al., 2009).

In anaerobic granules, the acetotrophic methanogenic archaeon *Methanosaeta*, which shows filamentous growth, can play an important role in biomass auto-aggregation (Wiegant, 1988; Tay et al., 2010; Li et al., 2015).

Anaerobic treatment of highly saline wastewater is of interest due to an increase in high salinity industrial or domestic waste streams (Xiao and Roberts, 2010). When using non-adapted sludge, methanogenic activity is inhibited and granules disintegrate with concomitant biomass wash-out, resulting in deterioration of process performance (Rinzema et al., 1988; Vallero et al., 2003; Ismail et al., 2008). Nevertheless, a start-up strategy with salinity-adapted biomass was successfully applied to overcome this issue (Feijoo et al., 1995; Hierholtzer and Akunna, 2014; Gagliano et al., 2017; Sudmalis et al., 2018).

Considering granules as aggregated biofilms held together by a specific matrix of EPS, the concentration and ratio of cations in the aqueous phase may influence their formation and structural integrity. Divalent cations such as Ca^2+^ favor the formation of compact biofilms, decreasing electrostatic repulsion during the initial stage of cell-to-cell adhesion, and forming bridges between anionic EPS molecules (Geesey et al., 2000; Flemming and Wingender, 2010).

In contrast, the role of monovalent Na^+^ in (bio)aggregation is controversial. The presence of high concentrations of monovalent cations such as Na^+^ could lead to displacement of the bridging divalent cations, preventing granule growth and having a negative effect on strength and stability of existing granules (Lambert et al., 1975; Bruus et al., 1992; Ismail et al., 2010). Higgins and Novak (1997) and Sobeck and Higgins (2002) showed a deterioration of bioaggregate properties (as size, volume, strength, charge, etc.) at high monovalent to divalent cation ratios. Nevertheless, the DLVO theory (Hermansson, 1999) states that Na^+^ will improve bioaggregation as the solution ionic strength increases and the surface charge of microbial cells decreases. Pevere et al. (2007) demonstrated that at increasing concentrations, Na^+^ was proportionally adsorbed by the granular sludge, without any negative effect on aggregation. The same was observed by Kara et al. (2008), who also demonstrated that Na^+^ addition led to a higher EPS production compared to a non-saline control.

Non-halophilic methanogenic Archaea, which are microbial key-players in granules formation, can accumulate Na^+^ at very high intracellular concentrations (Sprott and Jarrell, 1981; Scherer et al., 1983), because it is essential to generate an electrochemical ion gradient across the membrane for methane synthesis (Perski et al., 1982; Schlegel and Müller, 2013). Additionally, methanogenic Archaea are able to accumulate high concentrations of potassium under optimal conditions without any salt-stress (Schäfer et al., 1999; Roeßler and Müller, 2001), which is then increased as primary osmoprotectant when the cells are exposed to higher osmolality (Roeßler and Müller, 2001; Schlegel and Müller, 2011).

Upflow anaerobic sludge blanket reactors working at high salinity have only been studied in few works (e.g., Rinzema et al., 1988; Vallero et al., 2003; Ismail et al., 2008, 2010; Li et al., 2014; Aslan and Şekerdağ, 2015; Sudmalis et al., 2018). In a previous study (Gagliano et al., 2017), using a salinity-adapted inoculum, *Methanosaeta*-rich granules were formed in UASB reactors treating synthetic wastewater at 20 g/L Na^+^, achieving Chemical Oxygen Demand (COD) removal efficiencies of 94%.

In the present study, granule formation from the same salt adapted inoculum in two UASB reactors working at low (5 g/L Na^+^) and high (20 g/L Na^+^) salinity was investigated. Special focus was on glycoconjugate patterns of the EPS matrix within the granular biofilm by applying Fluorescence Lectin Bar-Coding (FLBC) (Neu and Kuhlicke, 2017). The lectin approach represents currently the only option for non-destructive, *in situ* glycoconjugate analysis and so far, this is the first study applying this method on anaerobic granules. Additional techniques including light, fluorescence and electron microscopy, together with the analysis of ion content and 16S rRNA genes, were applied to investigate the evolution of granules in time. The results indicate a key role of *Methanosaeta* in aggregation and in shaping EPS glycoconjugate patterns with the bacterial partners in response to salinity stress.

## Materials and Methods

### Reactors Operation

Two UASB reactors were inoculated with 6 g VSS/L of anaerobic sludge from a full-scale reactor treating acetic and benzoic acids rich saline wastewater (8 g Na^+^/L) from the Shell plant in Moerdijk, The Netherlands. The reactors were operated for 217 days at 35 ± 1°C, and fed with a synthetic wastewater consisting of macro and micro-nutrients (Sudmalis et al., 2018), containing glucose, acetate, and tryptone in a 3:2:1 COD ratio as a substrate. The concentration of the ions fed in both reactors was the same except for sodium. To compare the different salinity effects on the same inoculum, a low salinity reactor (LS) was operated at 5 g Na^+^/L, while a high salinity (HS) reactor was operated at 20 g Na^+^/L. The influent concentration increased in steps to reach a final organic loading rate (OLR) of 16 g COD/m^3^-d. A summary of operational conditions and performances is shown in **Table 1**.

**Table 1:** Upflow anaerobic sludge blanket (UASB) Reactors operation and main performances during the 217 days of process.

	LS Reactor	HS Reactor
*Operating Temperature*	35 ± 1°C	35 ± 1°C
*Start-up Inoculum*	6 g VSS/L	6 g VSS/L
*Salinity*	5 g Na^+^/L	20 g Na^+^/L
*Reactor volume prior sedimentation zone*	680 mL	730 mL
*Loading rate during start - up*	1 g COD/L⋅d	1 g COD/L⋅d
*Increase of influent COD from 3 g/L to 7 g/L^∗^*	Day 31	Day 31
*Increase of influent COD from 7 g/L to 12 g/L^∗^*	Day 52	Day 52
*Upflow velocity*	from 0.2 m/h to 1 m/h (day 30)	from 0.2 m/h to 1 m/h (day 30)
*Average Biogas Methane content*	63.6 ± 4.6%	68.4 ± 4.3%
*Average COD removal efficiencies*	97.2 ± 2.1%	94.0 ± 2.2%

^∗^*Increase of influent COD corresponds to increasing loading rates.*

*More details are reported in Sudmalis et al. (2018).*

### Inductively Coupled Plasma Optical Emission Spectroscopy (ICP-OES) Analysis

Fresh mixed liquor samples were collected from both reactors. Granular sludge was separated from the liquid phase by centrifugation at 10000 ×*g* at 4°C for 15 min. The supernatant was filtered through 0.2 μm membrane filters. Approximately 0.7 g of solids and 2.5 mL of supernatant were microwave digested (ETHOS 1 Labstation, Milestone S.r.l., Italy) adding 7.5 mL 37% HCl and 2.5 mL 65% HNO_3_. As a blank, Milli-Q water was treated in the same way. Diluted and digested samples were analyzed in duplicate by ICP-OES (Varian, Australia).

### DNA Extraction, Cloning and Sequencing

Genomic DNA was extracted from granules sampled at the end of the digestion time from both reactors using a FastDNA^®^ SPIN kit for soil (MPBio, United States) according to the manufacturer’s instructions. DNA concentration and purity were measured with a NanoDrop^®^ spectrophotometer (ThermoFisher Scientific, United States). PCR-amplicons of archaeal 16S rRNA genes were obtained using primers 25F (CYGGTYGATYCTGCCRG) and 1386R (GCGGTGTGTGCAAGGAGC) following the protocol of Borrel et al. (2012). Cloning of purified PCR products was performed using pGEM-T Easy Vector System into *Escherichia coli* JM109 competent cells (Promega, United States) according to the manufacturer’s instructions. A total of 95 clones from each reactor was selected for 16S rRNA amplicon sequencing and data analysis, as described in Gagliano et al. (2017). The sequences were deposited in GenBank and accession numbers are listed in Supplementary Table S1.

### Bright Field Microscopy and Crystal Violet Staining

Freshly sampled granules were periodically analyzed to check the aggregation process by bright field microscopy using a Leica EZ 4D Stereomicroscope equipped with a Coach DSC webcam (Leica microsystems, Germany). Crystal violet 0.1% (v/v) staining was used to visualize the EPS layer on granules (O’Toole et al., 1999).

### Scanning Electron Microscopy (SEM)

Granules taken from both reactors after 217 days were fixed with 2.5% (v/v) glutaraldehyde (in PBS/30% sacharose) for 15 h at 4°C and then in 1% (v/v) OsO_4_ (in PBS/30% sacharose) for 4 h at room temperature (RT). After fixation, granules were washed 3 times in PBS/30% sacharose (for 15 min). Then they were dehydrated in graded ethanol solutions (10, 30, 50, 70, 80, 90, 96, and 100%) for 10 min each. Finally, samples were air dried before analyzing with a Magellan 400 SEM (FEI Company, Hillsboro, OR, United States) at an acceleration voltage of 2 kV and beam current of 6 pA at RT.

### Fluorescence Microscopy Analyses

#### Fluorescent *in Situ* Hybridization (FISH) and Cryosectioning

At the end of the process granules from each of the UASB reactors were fixed with 37% formaldehyde (w/w) according to Amann et al. (1995). After fixation, samples were washed with PBS to remove the excess salinity, and then stored at −20°C in ethanol/ PBS (1:1) (Hugenholtz et al., 2002). FISH was carried out on granules gently crushed by flushing in 1 ml syringe with a 0.7 mm diameter needle. All oligonucleotide probes applied were labeled with Cy3-red or Alexa488-green fluorophores (listed in Supplementary Table S2). The larger and stronger HS granules (20 g/L Na^+^) were formaldehyde fixed and embedded in OCT compound (Sakura Finetek, Japan). Granules were cryosectioned (Microm HM 500OM Cryostat Microtome, Heidelberg, Germany) in slices of 10 μm and then used for FISH. OCT embedding and cryosectioning procedures are fully described in Batstone et al. (2004). For FISH fixation and OCT embedding a PBS/30% sacharose solution was used to preserve sodium content of the granules. To understand the possible association between cells and EPS domains, whole granules were treated with conventional FISH procedures in combination with the lectin staining (see next section). The ethanol graded series of dehydration steps were shortened from 3 to 1 min, avoiding damage of the EPS structures.

#### Fluorescence Staining of EPS Components

Fresh granules collected from LS and HS reactors were analyzed by FLBC (Neu and Kuhlicke, 2017) to assess the glycoconjugate pattern within the EPS matrix. The most significant lectins from the first screening, selected based on signal strength and structural domains stained, were used to characterize the EPS profiles in more detail by fluorescent lectin-binding analysis (FLBA). Lectins, either labeled with fluorescein isothiocyanate (FITC), Alexa488 or tetramethylrhodamine isothiocyanate (TRITC), were purchased from Sigma, EY Laboratories, Vector Laboratories, and Molecular Probes. Details of the applied lectins and their specificity are compiled in Supplementary Table S3. The lectin signal (specificity) was discussed using the suppliers’ data sheet. FITC and Sypro Orange (ThermoFisher Scientific, United States) were used to stain the protein portion of EPS. All stainings were carried out in the reactor liquid-phase for 30 min at RT in the dark. For microscopic observation, granules were washed with PBS/30% sacharose solution.

#### CoroNa Red Sodium Staining

For Na^+^ compartmentation analysis, sampled granules were placed in 500 μl PBS/30% sacharose solution. Then 1 μl of CoroNa Red sodium indicator (1 mM) (Thermo Fisher Scientific, United States) was added. The tubes were placed at 37°C for 3 h in the dark. In a final step granules were carefully washed before epifluorescence microscopy or Confocal Laser Scanning Microscopy (CLSM) analysis. To investigate the relation between sodium compartmentation and glycoconjugates, some CoroNa Red stained granules were further treated for 30 min at RT with the main positive lectins found after FLBC analysis.

#### Microscopy

Samples were examined either by epifluorescence microscopy (BX41, Olympus, Japan) equipped with Infinity Camera (Lumenera corporation, Canada), or by CLSM using a TCS SP5 (Leica, Wetzlar, Germany). CLSM datasets were recorded from granules in the multichannel mode taking advantage of reflection, F_420_ autofluorescence (Doddema and Vogels, 1978) as well as specific fluorochromes (details in Neu and Kuhlicke, 2017). The FIJI software package (version1.51g, Wayne Rasband, NIH, Bethesda, MD, United States) was used to merge epifluorescence image channels and to analyze/modify the CLSM stacks and channels. The software Imaris ver. 8.3.1 (Bitplane, Switzerland) was used for maximum intensity, isosurface and 3D projections of CLSM datasets.

## Results

### Reactors Operation and Granules Formation

**FIGURE 1:**
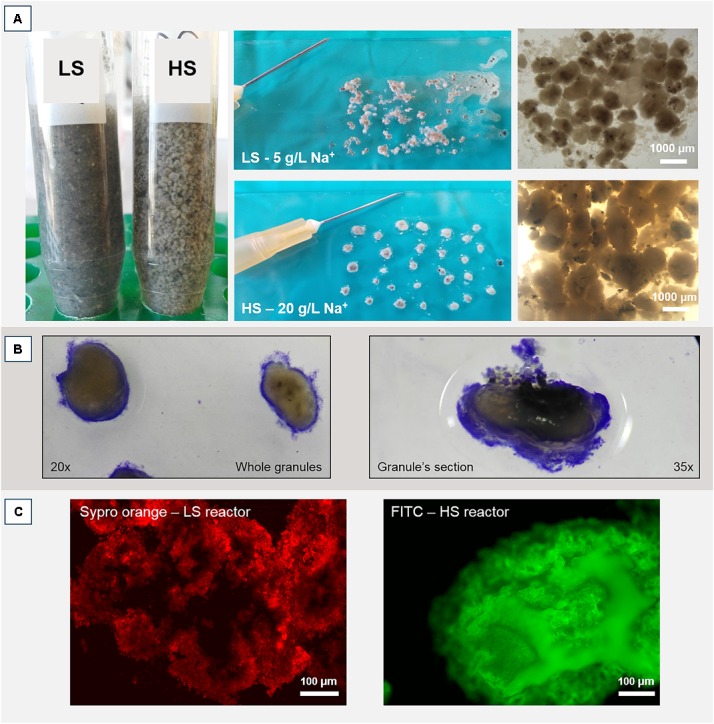
**(A)** Macroscopic and stereomicroscopic images of granules developed within the two UASB reactors working at 5 g/L of Na^+^ (LS) and 20 g/L of Na^+^ (HS) after 200 days of operation. **(B)** Crystal violet staining of a HS whole granule (left) and a HS sectioned granule (right). Please note the EPS layer surrounding the black core. **(C)** Protein fraction of the EPS layer contrasted by either FITC or Sypro Orange staining.

Operation and performance of the LS and HS UASB reactors, working at 5 and 20 g/L Na^+^ respectively, are presented in detail by Sudmalis et al. (2018). Both reactors produced methane throughout the 217 days operation regardless of the salinity level and this was accompanied by an efficient COD removal at increasing OLR (**Table 1**). In both reactors, the black colored particulate inoculum gradually aggregated into whitish granules (**Figure 1A** and Supplementary Figure S1). Microscopic analysis with crystal violet staining revealed a gel-like external layer (EPS) on a black core (**Figure 1B**). Fluorescence protein specific staining with FITC and Sypro Orange confirmed the presence of a complex polymeric layer on granules at both salinities (**Figure 1C**). The gel-layer was observed after 79 days of operation in granules grown at 20 g/L Na^+^, immediately after the increase of the OLR from 7 to 12 g/L COD.d (**Table 1**), while at 5 g/L Na^+^ it only started to appear after 135 days (Supplementary Figure S1). Granules grown in the HS reactor (20 g/L of Na^+^) were larger than those at lower salinity, and had a thicker EPS gel layer visible by naked-eye and microscopically (**Figure 1A**). These results suggest that Na^+^ did not have a negative effect on reactor performance, and even could have been beneficial for granule formation.

### Microbial Population Arrangement Within Granules

**FIGURE 2:**
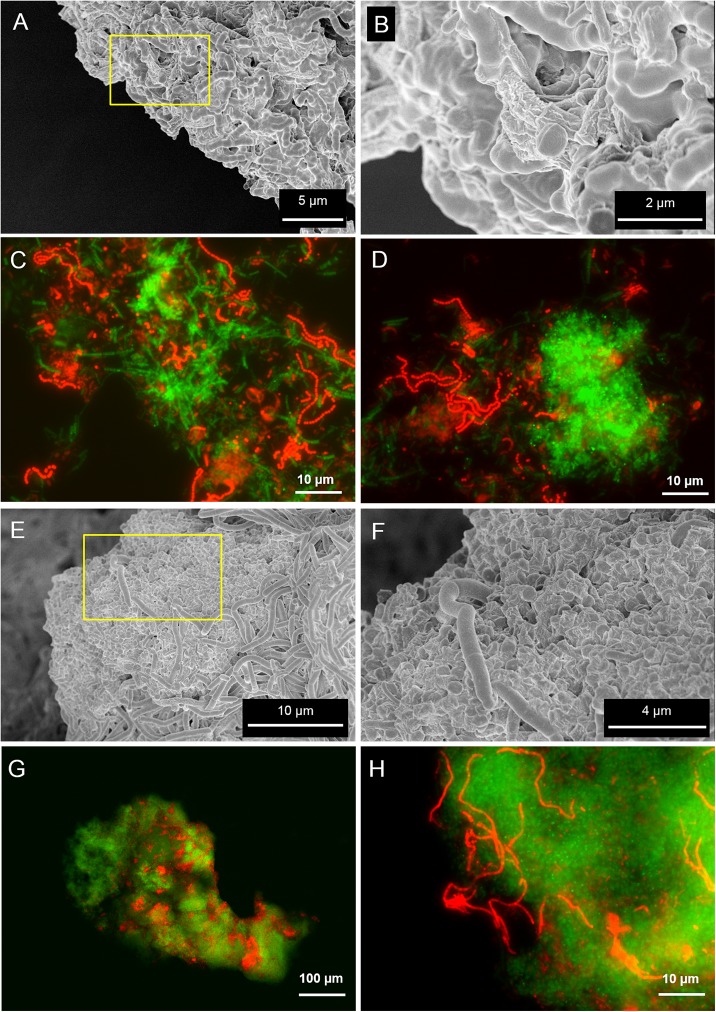
Scanning electron microscopy (SEM) and epifluorescence FISH images of samples taken from the LS reactor operated at 5 g/L of Na^+^ **(A–D)** and the HS reactor operated at 20 g/L of Na^+^ **(E–H)**. In **(B,F)**, SEM magnifications of the yellow insets indicated in **(A,E)**, respectively. The FISH images show in green the ARC915 probe (Archaea) and in red the EUB338 mix probes (Bacteria).

Scanning electron microscopy and fluorescence microscopy (FISH) analyses were applied to clarify the spatial organization of the microorganisms within the granules. SEM analysis revealed the presence of filamentous microbes in granules from both reactors (**Figures 2A,E**), although the morphology of the dominant microorganisms was different. FISH identification of the main microbial populations using the probes listed in Supplementary Table S2 showed that in the LS reactor the most abundant morphologies were twisted filamentous chains of cocci (**Figures 2A,B**). They were further identified as *Streptococcus* spp. (**Figures 2C,D**) with probe Strept. In HS granules two dominant morphologies were observed, both composed of rods aggregated as filaments (**Figures 2E,F**). Methanogenic Archaea, identified by FISH as the acetoclastic *Methanosaeta* spp., were the most abundant in both granules (**Figures 2C,D,G,H**, in green). *Methanosaeta* cells in both granule types were aggregated in two sorts of clusters: round shaped, rich in rods, and fibrous, with short filaments approaching each other (**Figures 2C,D** and Supplementary Figures S2A,B). In HS granules a rod/filamentous bacterium belonging to the family *Lachnospiraceae* (probe LAC435) was frequently found (**Figure 2H**). Probes Strept and LAC435 covered almost all the bacteria identified by the general probe EUB338 at 5 g/L Na^+^ and 20 g/L Na^+^, respectively (Sudmalis et al., 2018). This suggests an important role of these two bacteria in granulation, together with *Methanosaeta*, due to their filamentous morphology as highlighted in SEM images (**Figures 2A,E**). Both bacteria belong to the phylum *Firmicutes* and are reported as carbohydrate fermenters (Milinovich et al., 2008; Stackebrandt, 2014). Due to the abundance of the archaeal population, we confirmed the FISH results by clonal analysis of the same granule samples (Supplementary Table S1). Relatives of *Methanosaeta harundinacea* were identified as the dominant archaea in LS and HS granules, with 90 and 97% of the total clones, respectively. Just a few other identified and unidentified archaeal sequences were detected (Supplementary Table S1). Thus, different salinity promoted development of a different bacterial population, while *M. harundinacea* was the key methanogenic player at both salinities.

### Granules Cationic Content: ICP-OES Analysis and CoroNa Red Sodium Staining

Due to the importance of the balance between monovalent and divalent cations in bioaggregation and biofilm formation, their concentrations in (hydrated) granules and in the liquid fractions were determined by ICP-OES. The values listed in **Table 2** show that generally all the cations fed in the synthetic wastewater accumulated within the granules’ solid fraction. In the HS granules the concentration of Ca^2+^ was four times lower than in the LS granules. This difference indicates the displacement of divalent cations at increasing Na^+^ concentrations. On the other hand, the levels of divalent Mg^2+^ in both granules where way higher than in two liquid streams, but comparable to each other. Conversely, K^+^ accumulated within the HS granules to a very high concentration of 2.4 g/L, which is five times more than in the LS granules, mostly likely because of its role as osmoprotectant, especially in methanogens. The Na^+^ concentration in both LS (5 g/L Na^+^) and HS (20 g/L Na^+^) UASB was the same as in the liquid phase, suggesting that Na^+^ could have been retained either within cells and/or the EPS hydrated gel, without a negative effect on cell activity or biofilm structure. Fluorescence CoroNa Red-stained granules from LS and HS reactors are shown in Supplementary Figure S3. Epifluorescence microscopy showed that granules from HS reactor were strongly positive to the staining, while among the LS reactor granules there was much variability in fluorescence intensity (Supplementary Figures S3A,B). However, CLSM scans of several CoroNa Red stained granules revealed that fluorescence distribution throughout the structure had a variable alternation of positive and negative spots (Supplementary Figure S4). The positive spots constituted of the EPS layer and cells embedded within (Supplementary Figures S3C,D). Among cells, no major differences in the CoroNa red signal emission were retrieved when comparing the two samples. The highest signal originated from *Methanosaeta*-like cells (Supplementary Figure S3E), which may reflect an important role of Na^+^ in the energy metabolism of this methanogen. Overall, the results showed a high degree of Na^+^ retention in the HS granules coupled with Ca^2+^ displacement while the compact granule structure and integrity apparently were not compromised.

**Table 2:** Cations concentrations in the fed synthetic wastewater, hydrated granules and the reactor supernatant sampled from the sludge bed (bottom port) of the two UASB reactors.

	(mg/L)	Na^+^	K^+^	Ca^2+^	Mg^2+^
**LS reactor**					
*Synthetic wastewater*		5055.8	63.8	13.1	0.08
*Supernatant (sludge bed)*		5063.7 ± 92	70.4 ± 1	6.7 ± 0.3	5.4 ± 0.1
*Granules*		5752.4 ± 1892	422.8 ± 23.3	159 ± 63	78.6 ± 15.2
**HS reactor**					
*Synthetic wastewater*		20835	63.8	13.1	0.08
*Supernatant (sludge bed)*		20102.6 ± 206	–	7.9 ± 0.001	4.6 ± 0.3
*Granules*		22789 ± 1200	2370.5 ± 6.3	37.0 ± 36	99.9 ± 3.7

### Characterization of EPS Within Granules by Lectin Analysis

The EPS glycoconjugates were highlighted through FLBC screening by applying the commercial available lectins listed in Supplementary Table S3. The lectin patterns observed were classified in structural domains. Most of these structural domains were the same in LS and HS granules, but the lectin positive pattern was totally different, suggesting a different dynamics in the EPS glycoconjugate production with increasing salinity.

**FIGURE 3:**
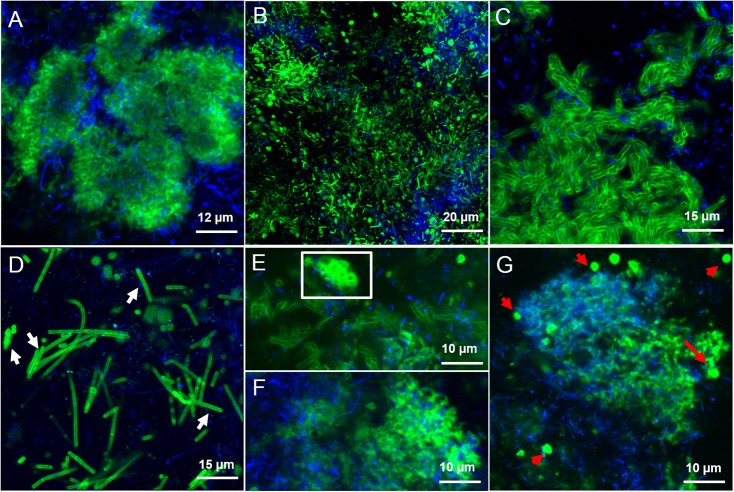
CLSM maximum intensity projections showing the extracellular glycoconjugate structures detected by FLBC analysis on LS reactor (5 g/L Na^+^) granules. In green the FITC lectin signal, in blue the F_420_ autofluorescence of methanogens. The lectins visualized are: **(A)** Ban, **(B)** AAL, **(C)** VRA, **(D)** Calsepa, **(E,F)** RPA, **(G)** PMA. In **(D)**, white arrows indicate *Streptococcus* chains. The white inset in **(E)** highlight the cloud-like EPS matrix diffusing from cell clusters. In **(G)**, red arrows are pointing at the EPS glycoconjugates vesicular structures.

In the LS granules the most relevant structure was a surface layer highlighted with BAN, HHA, PMA, and HAA lectins (an example in **Figure 3A**). The four binding profiles were similar, showing a thick outer layer within dense cell clusters, most likely rich in (α-1,3) and (α-1,6) linked Mannose (Man) structures (BAN, HHA, and PMA) and *N*-acetyl-Galactosamine (GalNAc) residues (bound by HAA). This surface layer was always identified in correspondence to the blue autofluorescence of methanogenic clusters, but the two signals never superimposed. A less thick surface layer was also identified with AAL, WFA, and WGA lectins (**Figure 3B**). It was mainly localized on non-aggregated rods or generally more dispersed cell clusters. AAL binds preferentially α-1,6-fucosylated oligosaccharides, but also Fucose (α −1,3) GalNAc groups. These GalNAc residues where then most likely highlighted by WFA and WGA.

**FIGURE 4:**
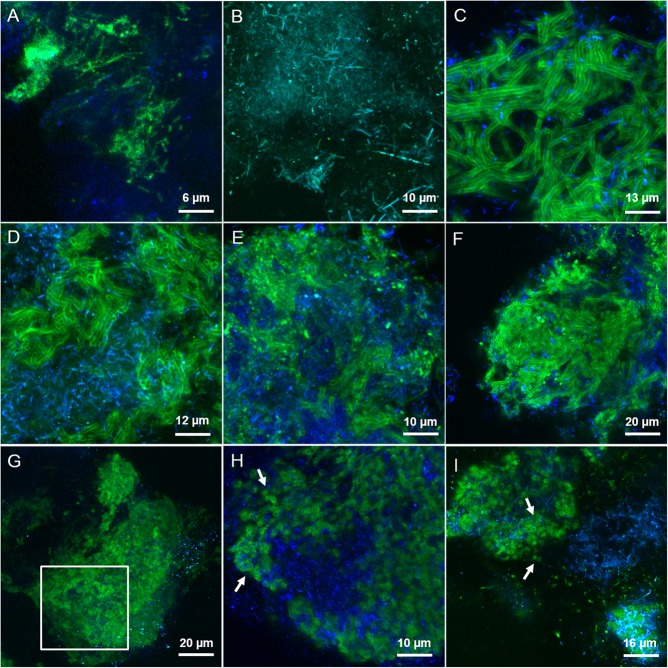
CLSM maximum intensity projections showing the extracellular glycoconjugate structures detected by FLBC analysis on HS reactor (20 g/L Na^+^) granules. In green the FITC lectin signal, in blue the F_420_ autofluorescence of methanogens. The lectins visualized are: **(A)** WFA, **(B)** GHA, **(C)** MOA, **(D)** LAL, **(E)** RPA, **(F)** SJA, **(G,H)** CAA, and **(I)** RCA-I. In **(H)**, a selected z-stack slice from a cropped region of the maximum projection intensity image shown in **(G)** (white inset). White arrows are pointing at small globular shaped subunits of the cloud-like matrix.

By applying the combination of WGA or AAL with HAA (labeled either in red or green) on the LS granules we concluded that most of the lectins were staining the same surface EPS glycoconjugates (yellow signal in Supplementary Figures S5A,B) on single rods and clusters of rods. Based on the microbial community analysis and cell morphology we speculate that *Methanosaeta* cells exhibit this complex glycoconjugate profile in LS granules. On the other hand, in the HS granules this type of surface EPS was rarely detected and only with WGA and WFA lectins (**Figure 4A**). The autofluorescent methanogenic clusters showed cell surface glycoconjugates detected by GHA, MPA, PNA and VGA lectins (**Figure 4B**). This indicates that under more stressful conditions (20 g/L Na^+^) the active *Methanosaeta* cells developed a glycoproteic layer with exposed α-linked Galactose (Gal) and GalNAc residues.

A complex capsular glycoconjugate structure was a dominant domain highlighted at both salinities. In the LS granules it was identified by ABA, ASA, MOA, RPA, SBA, VFA, and VRA lectins (**Figure 3C**). The glycoconjugate pattern most likely involved terminal Gal residues (MOA and VRA), (β-1,3) and (1,4) linkages between Gal and GalNAc (ABA, SBA, and RPA) as well as branched Man residues (ASA and VFA). The capsular glycoconjugates in the HS granules had a more diverse binding profile which was rich in Gal residues (positive to MOA lectin, **Figure 4C**) binding two different sugars: GalNAc through β [1-3(4)] bonds (identified with ECA, SBA, SJA, SSA, and VVA lectins) or Fucose (Fuc) by α (1-2) bonds (target of LAL, Lotus and LBA lectins) (representative examples in **Figures 4D–F**). In the HS granules, apart from MOA, all the lectins detecting the capsule resulted in a blurred signal (as seen in **Figures 4D,E**).

Another kind of capsular glycoconjugates in the LS granules was highlighted by the Man specific lectin Calsepa. This lectin was the only one found to stain the *Streptococcus* spp. cocci chains (**Figure 3D**, white arrows). None of the other lectins detected this peculiar capsule.

In the LS granules the most important lectin was RPA (specific for GalNAc rich glycosides), which detected all the structures cited before (**Figure 3E**). RPA was the only one together with PMA (Man specific lectin) to stain a diffuse glycoconjugate matrix mostly embedding the autofluorescent methanogenic clusters (**Figures 3F,G**). By applying the combination of PMA/WGA and RPA/HAA the connection between matrix and surface glycoconjugates could be highlighted (yellow signal in Supplementary Figures S5C,D). The PMA and RPA lectin highlighted the presence of several unusual structures with a globular, vesicle-like appearance (**Figure 3G**, red arrows). At a few locations the relatively large RPA blobs aggregated forming a continuous matrix as seen in **Figure 3E** (white inset).

**FIGURE 5:**
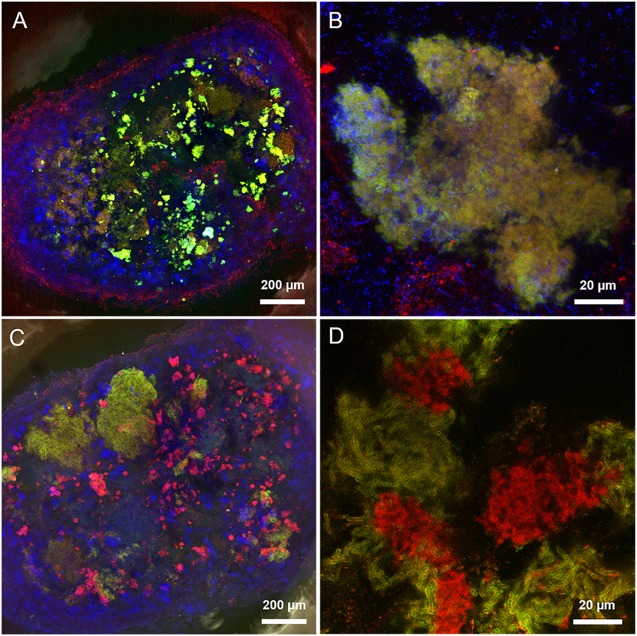
Maximum intensity projections of lectin combinations on high salinity granules. **(A,B)** PSA (in red) and CAA (in green). **(C)** LAL (in green) and PSA (in red). **(D)** PSA (in red) and MOA (in green). Yellow indicates co-localization of the two lectin signals. Autofluorescent methanogens are in blue.

The predominant structure highlighted by FLBC analysis in the HS granules was indeed a cloud-like diffuse granule matrix (**Figures 4G–I**). A closer look revealed small globular shaped subunits (indicated by white arrows in **Figures 4H,I**). This structural feature was aggregated into huge arrangements spread throughout the granule and always extensively embedding autofluorescent methanogenic clusters. The HS cloud-like EPS glycoconjugate was positive to CAA, RCA-I and PSA lectins. Their specificity indicates GalNAc, β-Gal and branched Man with Fuc as determinant. Lectin combinations applied on the HS granules further clarified the main role of this cloud-like structure. **Figures 5A,B** shows the distribution and shape of the cloud-like glycoconjugates through PSA (in red) and CAA (in green) lectins. Their signal is co-localized in most of the spots in the inner part of the granule (**Figure 5A**, in yellow), while only PSA stained the external layer of the granule (**Figures 5A,C**, in red). Moreover, the co-localization of the signal when combining PSA (red) with MOA (green) (**Figure 5D**), suggests a possible connection between the cloud-like and capsular structures specifically through Man and Gal residues. Indeed, when PSA was combined with other capsular glycoconjugate positive lectins, the co-localization of the signal was never observed (an example in **Figure 5C**). Overall, the development of the cloud-like EPS glycoconjugates, in fact the main difference between LS and HS granules, could be connected to larger granules obtained at high salinity along 217 days of the process.

### CoroNa Red and FISH Combined With Lectin Staining

**FIGURE 6:**
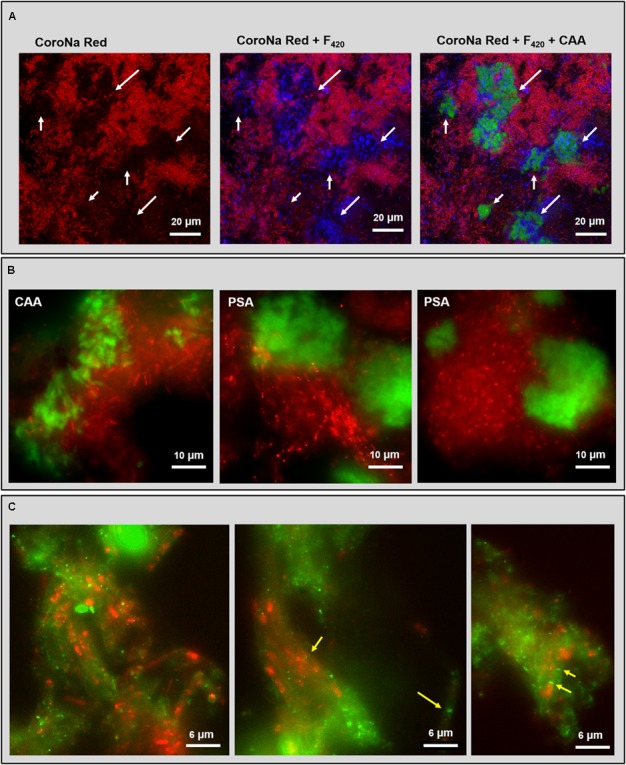
Fluorescent images of the HS reactor (20 g/L Na^+^) granules analyzed by combining lectin staining with either CoroNa Red sodium staining or FISH technique. **(A)** CLSM dataset showing the combination of CoroNa Red plus blue F_420_ autofluorescence and CAA lectin; images are showing the same location by adding individual channels. White arrows indicate the spots occupied by the cloudy EPS structure that were not positive to CoroNa Red. **(B)** Epifluorescence images of FISH (ARC915 probe in red) plus CAA and PSA lectin staining (in green). **(C)** Epifluorescence images of FISH (ARC915 probe in red) plus RPA lectin staining (in green); yellow arrows indicate the *Methanosaeta* spacer plugs within the capsule.

Some lectins detecting the cloud-like and capsular glycoconjugates in the HS (20 g/L Na^+^) granules were combined with either CoroNa Red staining or FISH to understand the relationship of these structural domains with the high Na^+^ content of these granules and the widespread methanogenic population. CoroNa Red staining showed Na^+^ distribution throughout most of the cells, as seen in Supplementary Figure S3. However, the active methanogenic cells embedded within the cloudy-EPS were not positive to the Na^+^ staining (**Figure 6A** and Supplementary Figure S6, white arrows), suggesting a protective role of the cloud-like EPS polymers for methane producing cells toward Na^+^ accumulation.

Among the several lectins tested together with CoroNa Red, only the PSA signal at some points overlapped with the red signal on cell surfaces and within the cloud-like glycoconjugates (Supplementary Figure S6), suggesting an extracellular co-localization of Na^+^ and Man. Cells covered by capsular glycoconjugates were positive to CoroNa Red staining in many spots (Supplementary Figure S7).

PSA and CAA lectin staining in combination with the domain specific probe ARC915 often showed an association between *Methanosaeta* clusters and cloud-like glycoconjugates (**Figure 6B**), both in rounded and short filaments conformations (**Figure 6B** with PSA). The CAA signal showed a more “slimy” appearance than the PSA one (**Figure 6B**), suggesting an embedding function for the Gal/GalNAc rich portion of the cloud rather than the for Man rich portion, which looks more superficial.

The combination of RPA with ARC915 showed the association of capsular glycoconjugates and *Methanosaeta* cells when aggregated in short filaments clusters (**Figure 6C**). This structure looks similar to the one described by Beveridge et al. (1986) in *M. concilii*, where the filament is surrounded by a tubular capsule and each cell is separated from the next by a spacer plug (**Figure 6C**, yellow arrows). Interestingly, no capsule was observed on *Methanosaeta* cells when aggregated in round clusters. In many capsular EPS spots no FISH signal was detected, which may have been caused by a shorter ethanol dehydration step in the FISH procedure, causing a limited probe penetration (see M&M).

## Discussion

This microscopy study is the first one that investigated the effect of Na^+^ on anaerobic granule formation, with special emphasis on EPS glycoconjugate profiles. The granules in both UASB reactors operated at 5 and 20 g/L Na^+^ contained a high density of EPS producing cells and accumulated very high amounts of Na^+^, which was non-uniformly distributed in the aggregates. Our results support the Na^+^ biosorption theory and its positive effect on EPS production and bioaggregation (Hermansson, 1999; Pevere et al., 2007; Kara et al., 2008). Thus, Na^+^ is maybe the driving force to get a higher production of EPS glycoconjugates at high salinity if compared to low salinity (**Figure 1**). Due to the dominance and persistence of *M. harundinacea* in the shift from low to high salinity, and the presence of the same structural domains in both granules, this methanogen could be a protagonist in shaping the different arrangements of the glycoconjugates and the excretion of the cloud-like matrix.

### Sodium Localization and the Cloud-EPS Role

**FIGURE 7:**
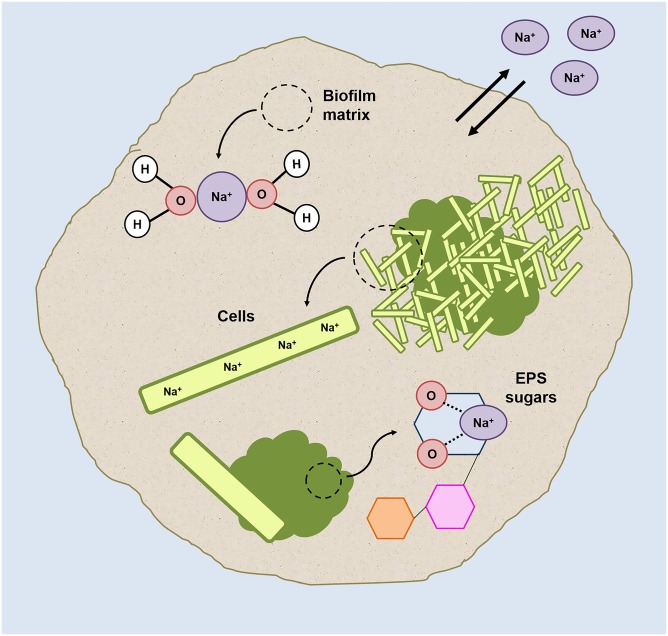
Graphic representation of the possible sodium localizations within the HS granules (grown at 20 g/L Na^+^) on the basis of the results collected in this study.

A graphical resume of sodium localization in HS granules is shown in **Figure 7**. Because high intracellular Na^+^ levels would have a lytic effect (Roeßler and Müller, 2001), the high Na^+^ concentration at HS can be explained mostly by diffusion into the granule matrix (which behaves as an aqueous solution, as explained in Stewart and Franklin, 2008) rather than by intracellular accumulation.

The significant differences in Ca^2+^ concentration between HS and LS granules (**Table 2**) confirms divalent cation displacement by Na^+^, most likely by ion-exchange of the gel-forming polysaccharides. The displacement phenomenon didn’t have any detrimental effect on the integrity of the granules’ EPS (Supplementary Figure S1), as Na^+^ ions can bind to oxygen atoms of several carbohydrates (Heaton and Armentrout, 2008; Mayes et al., 2014), and the affinity of this binding could even become higher at increasing sodium concentrations (Wang et al., 2015).

Our hypothesis is that, to keep the aggregation ongoing and to protect inner cells, in HS granules part of the Na^+^ is directly complexed and “inactivated” with the cloud-like EPS matrix components. The granules’ EPS profiles shift from 5 to 20 g/L Na^+^, and especially the cloudy-EPS glycoconjugate matrix growth and its enrichment in Gal and Man, could reflect the need for different cations/polysaccharides interactions to protect part of the cells from Na^+^ accumulation. Mannose-rich EPS was present throughout the HS granules especially in the external gel layer (**Figures 6A,C**). The co-localization of Man residues and sodium in some regions of the HS granules (Supplementary Figure S6) further supports our hypothesis.

Hecht and Srebnik (2016) recently showed that when alginate is enriched in poly-mannuronate residues (an oxidized form of Mannose), is more prone to form stable polymer chains in presence of Na^+^ ions. Stewart et al. (2014) proposed that Na^+^ interacts with the oxygens of the mannuronate molecule, but not with the carboxylate portion of polysaccharide chains. Thus, gel formation is not inhibited by Na^+^, but its 3D structure could be weaker than the one formed by complexing with Ca^2+^.

### *M. harundinacea* and the Glycoconjugate Structural Domains

FLBA granules’ profiles and FISH/Lectin staining assigned the microbial origin of capsular and cloudy-EPS to *Methanosaeta* cells (**Figures 6B,C**), the dominant microorganism at both salinities (**Figure 2**). Two types of *Methanosaeta* aggregations were highlighted by FISH (Supplementary Figure S2B) showing a different behavior in terms of EPS coverage. The two shapes of clusters (round and fibrous) could represent different subspecies of *M. harundinacea* exploiting different functions, due to the range (97–99%) of identity observed within clones (Supplementary Table S1).

**FIGURE 8:**
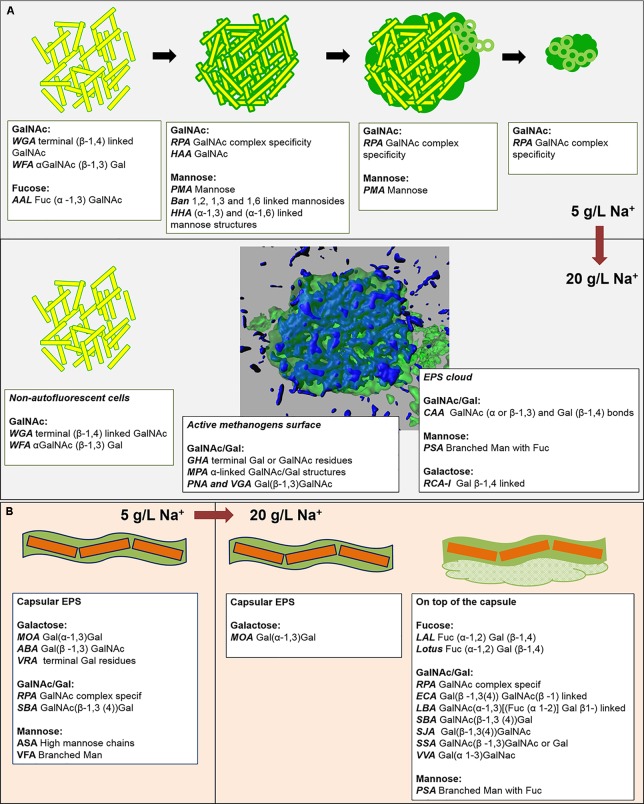
**(A,B)** Graphical summary of the main EPS profiles of the glycoconjugate domains detected by FLBC and FLBA analyses, and their changes with the increase of salinity from 5 to 20 g/L of sodium. The lower half of **(A)** contains a 3D isosurface view of a CLSM maximum-intensity projection of the cloud-like EPS glycoconjugates (green) embedding autofluorescent methanogens (blue).

A linkage between the surface layer on the *Methanosaeta*-like round clusters and the excreted matrix at low salinity was becoming clear by lectin combinations (**Figure 8A** and Supplementary Figure S5). At high salinity, this layer almost disappeared, while the active methanogens developed other surface glycoconjugates rich in Gal(β-1,3)GalNAc bonds and GalNAc/Gal structures. The surface-EPS glycoconjugates could be connected with the GalNAc and Gal residues of the protective cloud-like glycoconjugates (**Figure 8A**). In this case, it appears to be released mostly onto cells surfaces with no visible means of attachment.

In HS granules a possible association between cloudy and capsular EPS glycoconjugates was suggested by the continuity when applying the combination of PSA and MOA lectins (yellow signal in **Figure 5D**). The thick capsule covered the short filamentous clusters of *Methanosaeta* (**Figure 6B**), which were the most common morphology identified in all the CoroNa Red tests (like in Supplementary Figure S3D). As shown in **Figure 8B**, the salinity increase from 5 to 20 g/L of Na^+^ changed the glycoside profile of the capsule, indicating an additional layer showing a complex Gal/GalNAc positivity plus the Fuc appearance. Fuc is an important sugar within the HS capsule structure, because LAL, Lotus and LBA positivity highlighted the connection of this deoxy sugar with GalNAc and Gal residues. Fuc identification seems to be a further proof of the connection between the capsule and the cloud, as it is a crucial determinant in the recruitment of branched Man structures by the PSA lectin protein domain (Kornfeld et al., 1981).

The ability of microorganisms to change the pattern of external glycoconjugates in response to changes in salinity was shown in previous studies using pure cultures (Baumgarten et al., 2012; Philips et al., 2017). The halophilic archaeon *Haloferax volcanii*, which forms Man rich biofilms under optimal growth conditions (Chimileski et al., 2014), modifies by N-glycosylation the composition of its surface layer glycoproteins when salinity decreases (Guan et al., 2012). Different from monocultures, in UASB systems a multitude of microbial species participate and contribute to the granule properties. Since the central carbohydrate metabolism of methanogens lacks some classical pathways that generate most of the sugar residues detected in this study (Bräsen et al., 2014), it is likely that the bacterial partners play a key role in “cross-feeding” *Methanosaeta*. The switch in the EPS pattern and eventually the cloud-like EPS excretion machinery could be the result of this partnership. Nevertheless, the salt stress response of *M. harundinacea* requires further study.

### *M. harundinacea* Coping With Salinity Stress

EPS/biofilm production and halotolerance from pure cultures of *M. harundinacea* species members were never reported. However, by analyzing *in silico* the complete genome of *M. harundinacea* strain 6Ac (Zhu et al., 2012) we could localize several genetic loci which products are potentially involved in the production of surface glycoconjugates and the synthesis of osmoprotectants. The methodology of reconstruction of the genetic loci is described in Supplementary Material.

First of all, *M. harundinacea* 6Ac genome includes a gene cluster containing all the components of an *N*-glycosylation pathway (Supplementary Figure S8) resembling the salinity-sensitive mechanism found in *H. volcanii* (Guan et al., 2012). Besides the cluster, which contains 20 coding regions, some external loci encode for multiple copies of the olygosaccaryltransferase AglB, the enzyme responsible for the final delivery of the assembled glycans on the cell outer layer. As cited in a previous phylogenetic study on archaeal *N*-glycosylation by Kaminski et al. (2013), the presence of multiple AglB sequences in a single species could be a reflection of the existence of enzymes with different specificities, which represents a microbial strategy for the addition of different glycans in function of the local growth conditions (Kaminski et al., 2013).

Secondly, this microorganism possesses a series of genes which products are most likely involved in the pathways for the synthesis of ectoine and N^𝜀^-acetyl-β-lysine from aspartate (Supplementary Figure S8). Both compounds have an osmoprotective function and they are produced and accumulated only under high salinity stress. However, while the effective ectoine biosynthesis, which is “typical” bacterial, has been detected recently in the Thaumarchaeon *Nitrosopumilus maritimus* (Widderich et al., 2016), the most rare N^𝜀^-acetyl-β-lysine production is restricted to methanogenic archaea (Pflüger et al., 2003) and some halophilic bacteria (Joghee and Jayaraman, 2014).

These genomic features, together with the structural filamentous behavior, apparently makes *M. harundinacea* the “perfect microbe” able to drive granulation and maintain it even under salinity stress. This emerges considering the results collected in this study and the previous evidences about the potential of *M. harundinacea* to improve sludge granulation in UASB reactors both in normal (Li et al., 2015) and elevated salinity levels (Gagliano et al., 2017; Sudmalis et al., 2018). Further insights into the protein expression patterns could confirm the powerful role of *M. harundinacea* within microbial communities applied for high salinity wastewater treatment through UASB systems.

## Author Contributions

MG, DS, HT, and CP conceived the study. DS designed, operated and monitored the laboratory-scale bioreactors, and conducted the ICP-OES analysis. MG, TN, and UK designed and operated the FLBC experiments, the confocal microscopy procedure and the microscopy image analysis. MG designed and operated the FISH and FISH-lectin approach plus the CoroNa Red experiments, and prepared the sequencing libraries. MG, TN, UK, and DS interpreted the results. MG drafted the paper. CP, HT, MG, TN, and DS revised the document. All authors read and approved the final manuscript.

## Conflict of Interest Statement

The authors declare that the research was conducted in the absence of any commercial or financial relationships that could be construed as a potential conflict of interest.
