# Supplementary material for: EPS Glycoconjugate Profiles Shift as Adaptive Response in Anaerobic Microbial Granulation at High Salinity
Source: Front Microbiol. 2018 Jul 2;9:1423. doi: 10.3389/fmicb.2018.01423 (PMC6036115; doi:10.3389/fmicb.2018.01423)
Supplement: Supplementary file 1 [file Data_Sheet_1.PDF]

Supplementary Material

EPS glycoconjugate profiles shift as adaptive response in anaerobic microbial granulation at high salinity

Maria Cristina Gagliano [*], Thomas R. Neu, Ute Kuhlicke, Dainis Sudmalis, Hardy Temmink and Caroline M. Plugge

*** Correspondence:** Dr. M.Cristina Gagliano: cristina.gagliano@wetsus.nl

Table S1 – Archaeal 16S rRNA gene clonal analysis of LS and HS UASB reactors after 217 days of digestion process.

LS reactor

**no. of clones**	**Accession number**	**Affiliation**	**Closest relative (accession no.)**	**Similarity (%)**
85	MG062723	*Uncultured Methanosaeta*	*Methanosaeta harundinacea 8Ac (NR_043203.1)*	97-99
1	MG062729	*Methanosaeta concilii*	*Methanosaeta concilii strain GP6 (NR_102903.1)*	99
3	MG062727	*Uncultured Methanolinea*	*Methanolinea tarda strain NOBI-1 (NR_028163.1)*	96
1	MG062726	*Uncultured Methanobacteriales*	*Methanobrevibacter ruminantium (NR_074117.1)*	84
4	MG062728	*Uncultured Methanobacteriales*	*Methanobacterium oryzae strain FPi 1 (NR_028171.1)*	77
1	MG062725	*Uncultured Thermoplasmata*	*Methanomassiliicoccus luminyensis strain B10 (NR_118098.1)*	92
95				

HS reactor

**no. of clones**	**Accession number**	**Affiliation**	**Closest relative (accession no.)**	**Similarity (%)**
92	MG062730	*Uncultured Methanosaeta*	*Methanosaeta harundinacea 8Ac (NR_043203.1)*	97-98
3	MG062724	*Uncultured Methanococcales*	*Methanothermococcus okinawensis strain IH1 (NR_102915.1)*	84
95				

Table S2 - Oligonucleotide probe sequences, target microbial groups and stringency conditions used in this study.

Name	Target group	Probe sequence (5'-3')	Formamide (%)	Reference
**ARCH915**	*Archaea*	GTGCTCCCCCGCCAATTCCT	20 or 35	Stahl and Amann, 1991
**EUB338, EUB338-II, EUB338-III**	*Bacteria*	GCTGCCTCCCGTAGGAGT, GCAGCCACCCGTAGGTGT, GCTGCCACCCGTAGGTGT	20 or 35	Amann et al., 1990
**BET42a**	*Betaproteobacteria*	GCCTTCCCACTTCGTTT	35	Manz et al., 1992
**GAM42a**	*Gammaproteobacteria*	GCCTTCCCACATCGTTT	35	Manz et al., 1992
**ALF968**	*Alphaproteobacteria*	GGTAAGGTTCTGCGCGTT	20	Neef, 1997
**CF319a**	*Bacteroidetes*	TGGTCCGTGTCTCAGTAC	35	Manz et al., 1996
**DELTA485A, DELTA495B, DELTA495C**	*Deltaproteobacteria*	AGTTAGCCGGTGCTTCCT, AGTTAGCCGGCGCTTCCT, AATTAGCCGGTGCTTCCT	35	Lücker et al., 2007
**CFX1223**	*phylum Chloroflexi*	CCATTGTAGCGTGTGTGTMG	35	Björnsson et al., 2002
**SYN961**	*Synergystetes*	GTTCTTCGGTTTGCATCG	20 or 35	Gagliano et al., 2015
**NLIMI91**	*Nostocoida Liimicola type I (Thricococcus spp)*	CGCCACTATCTTCTCAGT	35	Liu and Seviour, 2001
**STREPT**	*Streptococcaceae*	CACTCTCCCCTTCTGCAC	30	Trebesius et al., 2000
**LAC435**	*family Lachnospiraceae*	TCTTCCCTGCTGATAGA	35	Kong et al., 2010
**MSMX860**	*Methanosarcinales**	GGCTCGCTTCACGGCTTCCCT	45	Raskin et al., 1994
**MX825**	*Methanosaetaceae***	TCGCACCGTGGCCGACACCTAGC	50	Raskin et al., 1994
**MX825b**	*Methanosaetaceae subgroup***	TCGCACCGTTGCCGACACCTAGC	50	Crocetti et al., 2006
**MX825c**	*Methanosaetaceae subgroup***	TCGCACCGTGGCTGACACCTAGC	50	Crocetti et al., 2006
**MG1200b**	*most Methanomicrobiales*	CRGATAATTCGGGGCATGCTG	20	Crocetti et al., 2006
**MB311**	*Methanobacteriales*	ACCTTGTCTCAGGTTCCATCTCC	30	Crocetti et al., 2006

* (including *M.harundinacea*)
** (except *M.harundinacea*)

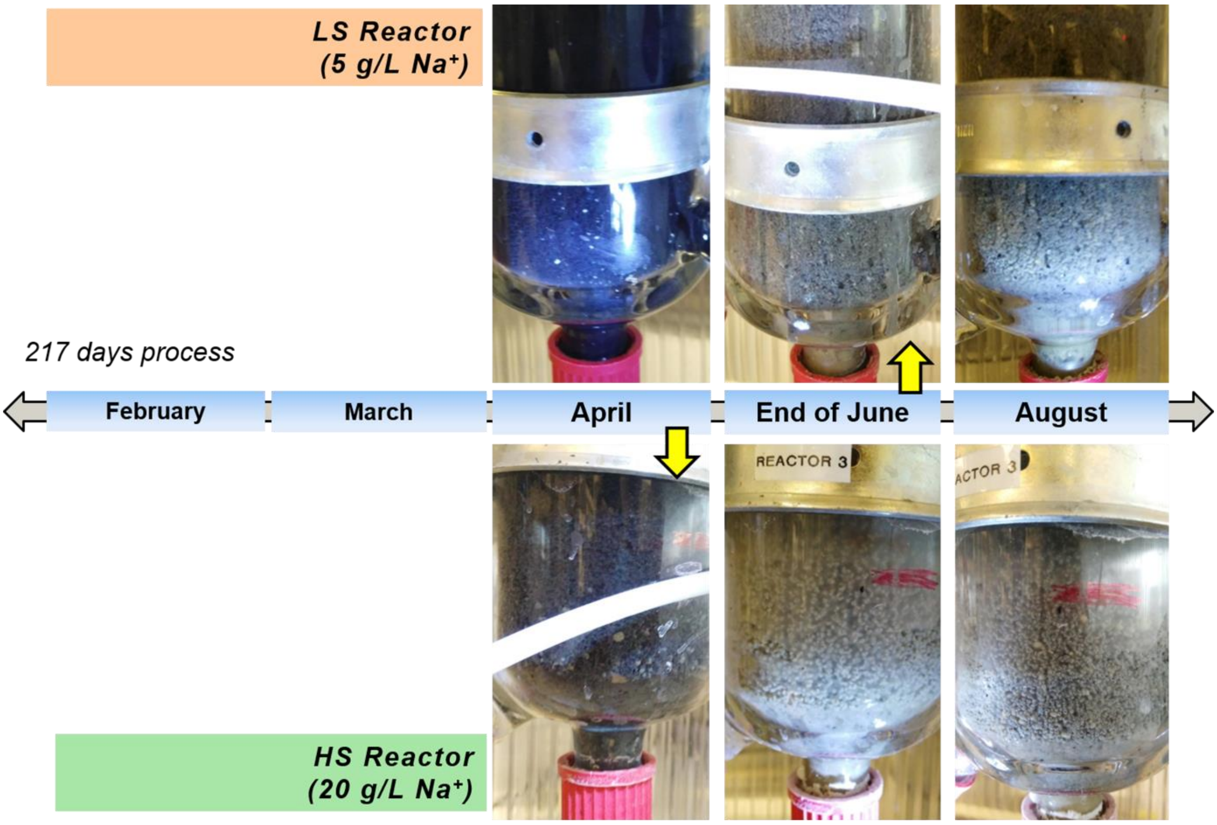

Fig. S1 – Granules development during time within the two UASB reactors working at 5 g/L of Na^+ (LS) and 20 g/L of Na^+ (HS) along 217 days of operation. Yellow arrows indicate the time period at which formed granules started to be clearly visible within the two reactors.

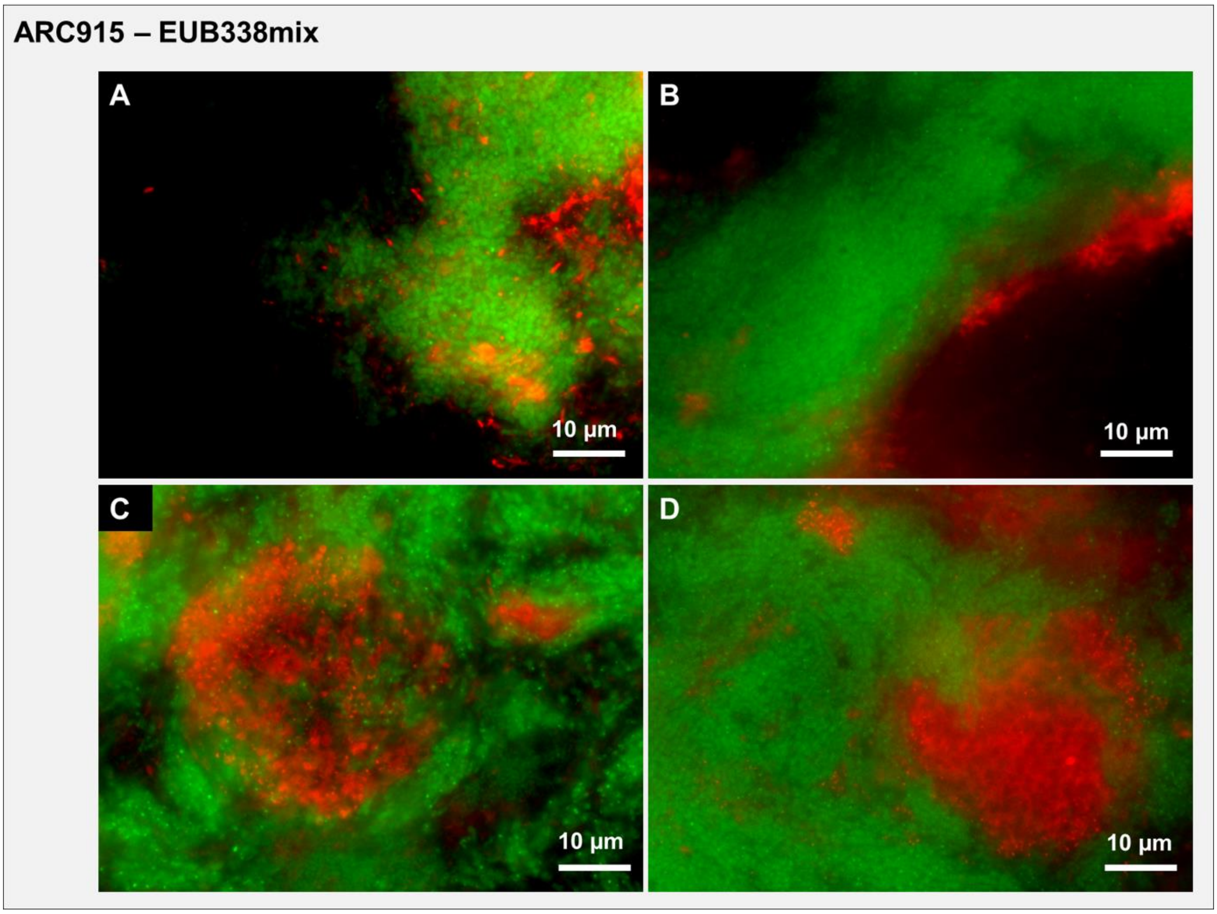

Fig.S2 – Epifluorescence images after FISH analysis of 10 μm thin section from a high salinity granule. In green ARC915 probe (Archaea), in red EUB338 mix probes (Bacteria).

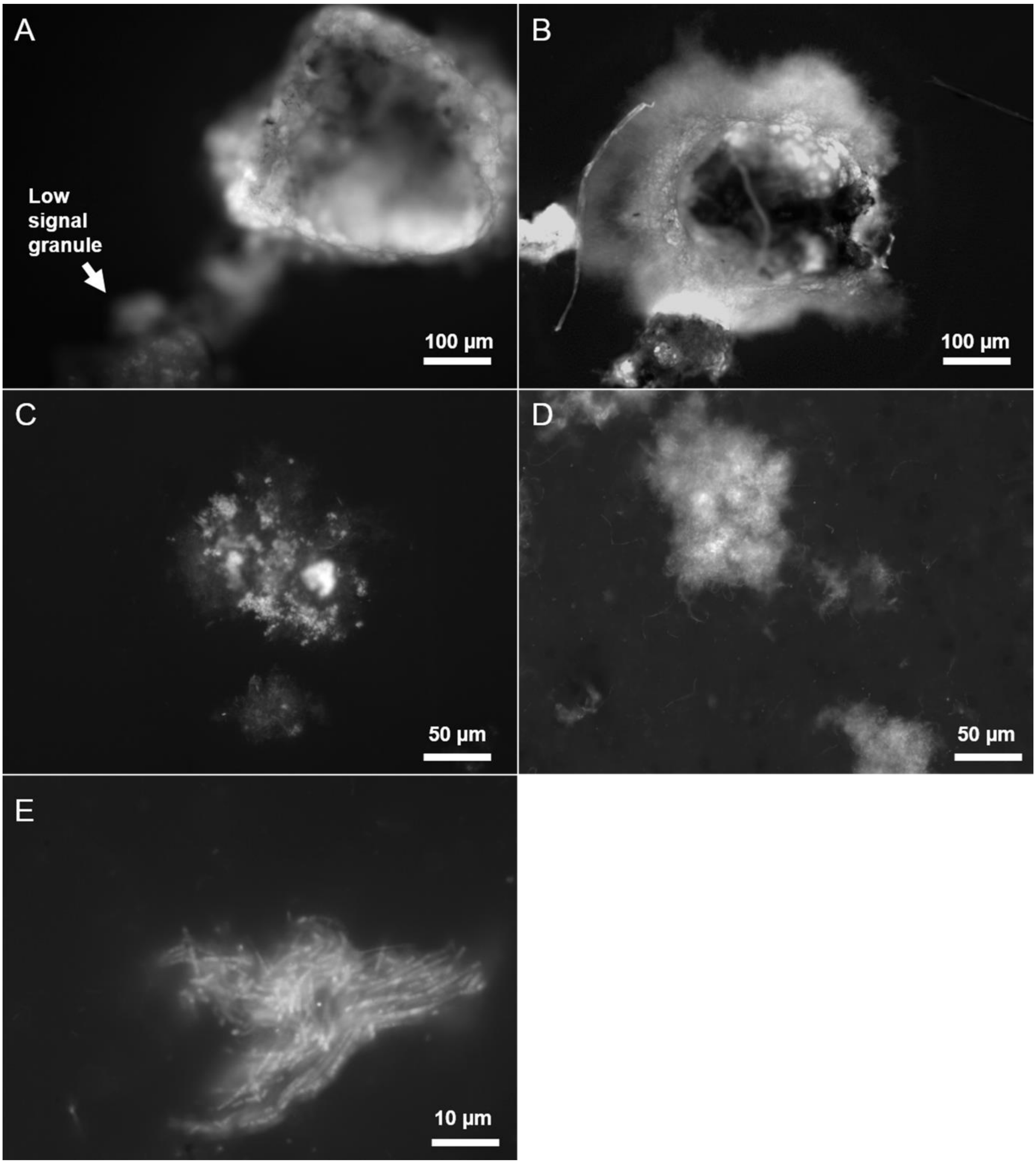

Fig. S3 - Epifluorescence of CoroNa Red sodium staining of LS (A and C) and HS (B and D) reactors granules. In E, a positive *Methanosaeta* cluster. Take notice of CoroNa Red staining localized sodium within the *Methanosaeta*-like cells, because of its important role in methanogenesis and other cell-transport activities. In particular, *Methanosarcinales*, that possess cytochromes across the membrane, are able to translocate more ions than the other methanogens (Schlegel and Müller, 2013).

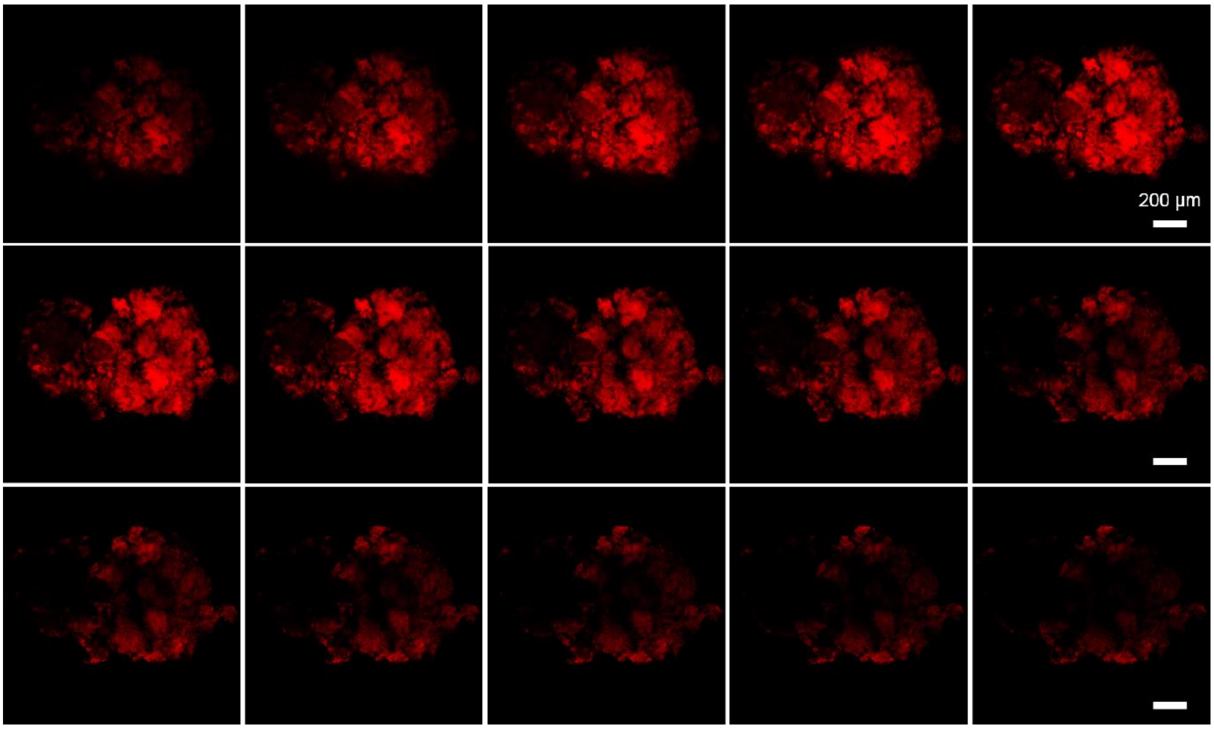

Fig. S4 – CLSM image data stack shown as gallery view. The CoroNa Red stained HS granule sample was recorded with a 3 µm step size.

Table S3 – Detailed overview of the lectins applied in the FLBC screening

Nr.	FITC conjugated Lectins (Abbreviation_latin name)	Single-sugar binding specificity	Carbohydrates linkage type	References (*)
1	AAA_Anguilla anguilla	α-Fucose	Fuc(α -1,3) Gal or L-Fuc	Gercken and Renwrantz, 1994
2	AAL_Aleuria aurantia	α-Fucose	Fuc(α -1,6)GlcNAc or Fuc(α -1,3) GalNAc related structures	Kochibe and Furukawa, 1980
3	ABA_Agaricus bisporus	Galactose	Gal(β -1,3) GalNAc, Gal(β -1,4) Glu	Presant et al., 1972; Nakamura-Tsuruta et al., 2006
4	ACA_Amaranthus caudatus	Galactose	Gal(β -1,3) GalNAc	Rinderle et al., 1989
5	AIA_Artocarpus integrifolia	Galactose	Gal(β -1,3) GalNAc, Gal(β -1,4) GalNAc, Gal(β -1,4) Glu	Sastry and Surolia,1986
6	AMA_Arum maculatum	Not inhibited	(Galβ1,4GlcNAc)	Allen, 1995
7	ASA_Allium sativum	Mannose	High mannose chains, D-Man(α -1,3)	Dam et al., 1998
8	Ban_Musa paradisiaca	D-Mannose and D-Glucose	1–2, 1–3, and 1–6 linked mannosides/glucosides	Singh et al., 2005
9	BDA_Bryonia dioica	N-Acetylgalactosamine	not determined	Peumans et al., 1984
10	BPA_Bauhinia purpurea	β D-galactose	Gal (β- 1,3) GalNAc	Yamamoto et al., 1992
11	CA_Colchicum autumnale	Lac>GalNAc>Gal and related sugars	Gal (β-1,4) Glu,	(*)
12	CAA_Caragana aborescens	N-Acetylgalactosamine and Galactose	GalNAc (α- 1,3) GalNAc, Galβ1→4GlcNAc , multi-valent Gal (β -1,4) GlcNAc and cluster forms of GalNAc (α 1- Ser/Thr)	Bloch et al., 1976; Wu et al., 1999

13	Calsepa_Calystega sepiem	Mannose and Maltose	α- D - mannopyranoside, methyl α- D - glucopyranoside	Nakamura-Tsuruta et al., 2008
14	ConA_Canavalia ensiformis	α-Mannose and Glucose	α-linked branched Mannopyranoside	Hardman and Ainsworth, 1976; Gupta et al., 1996
15	CPA_Cicer arietinum	Not inhibited	Not determined	(*)
16	CSA_Cytisus sessilifolius	Galactose and Lactose	GlcNAc oligomers	(*)
17	DBA_Dolichos biflorus	α-N-Acetylgalactosamine	GalNAc (α- 1,3) GalNAc	Piller et al., 1990
18	DGL_Dioclea grandiflora	Mannose and Glucose	α- D - mannopyranoside, Asn-linked carbohydrates	Gupta et al., 1996
19	DSA_Datura stramonium	N-acetylglucosamine	GlcNAc (β -1,4) GlcNAc	Yamashita et al.,1987; Kawashima et al., 1990
20	ECA_Erythrina cristagalli	Galactose, N-Acetylgalactosamine, Lactose	Gal(β -1,3(4)) GalNAc(β -1) linked	Berman et al., 1985
21	EEA_Euonymus europaeus	Not inhibited	Gal (α1-3)[(Fuc (α1-2)]Gal (β1-4) GlcNAc	(*)
22	GHA_Glechoma hederacea	N-Acetylgalactosamine/Galactose	O-glycans with exposed terminal Gal or GalNAc residues	Wang et al., 2003
23	GNA_Galanthus nivalis	Mannose	Terminal Man (α1-3) Man in olygomannosides	Shibuya et al., 1988
24	GS-I_Griffonia simplicifolia	Galactose, N-Acetylgalactosamine	GalNAc (α- 1,3) GalNAc, GalNAc (α- 1,3) Gal	Shibata et al., 1982
25	HAA_Helix aspersa	N-Acetylgalactosamine	Not determined	Fountain and Campbell,1984
26	HHA_Hippeastrum hybrid (Amaryllis)	Mannose	(α-1,3) and (α-1,6) linked mannose structures	Kaku et al., 1990
27	HMA_Homarus americanus	N-Acetylglucosamine	Not determined	Battison and Summerfield, 2009.

28	HPA_Helix pomatia	N-Acetylgalactosamine	GalNAc (α- 1,3) GalNAc, Gal(α-1,3)GalNAc	Piller et al., 1990
29	IRA_Iris hybrid	N-Acetyl-D-Galactosamine.	Not determined	(*)
30	LAA_Laburnum alpinum	N-Acetylglucosamine	GlcNAc (β -1,4) GlcNAc	(*)
31	LAL_Laburnum anagyroides	L-Fucose	Fuc (α 1-2) Gal (β 1-4) Glu and Fuc (α 1-2) Gal (β 1-4) Glc (β 1-6) GalNAc (β 1- 3)Gal	Piskarev et al., 2007
32	LBA_Phaseolus lunatus	N-acetylgalactosamine	GalNAc (α1-3)[(Fuc (α1-2)]Gal (β1-) linked	(*)
33	LcH_Lens culinaris	Mannose, Glucose	Branched Mannose structures with Fucose as determinant	Debray, H., Montreuil, J., 1983
34	LEA_Lycopersicon esculentum	N-acetyl-β-D-glucosamine	GlcNAc (β -1,3) GlcNAc, Gal(β -1,3(4)) GalNAc(β -1) linked	Kawashima et al., 1990
35	LFA_Limax flavus	N-acetyl and N-glycolyl neuraminic acid, Sialic Acid	α-Neu5Acp or sialic acid in any linkage to the subterminal sugar	Knibbsso et al., 1993
36	Lotus_Lotus tetragonolobus	α-Fucose	Fuc (α 1-2) Gal (β 1-4) [(Fuc (α1-2)] GlcNAc	Pereira and Kabat, 1974
37	LPA_Limulus polyphemus	N-acetyl and N-glycolyl neuraminic acid, Sialic Acid	not determined	(*)
38	MAA_Maackia amurensis	Sialic Acid	α-sialyl-[2→3]-lactose	Geisler and Jarvis, 2011
39	MNA-G_Morniga G	GalNAc/Gal ≫ Man/Glc	GalNAc(β 1-3)Gal (P)	(*)

40	MOA_Marasmium oreades	Not inhibited	Gal(α 1–3)Gal terminating carbohydrates and Gal(α 1–3)Gal(β 1–4)GlcNAc(β 1-) and Galα(1,3)[Fucα(1,2)]Gal	Kirkeby et al., 2004
41	MPA_Maclura pomifera	N-Acetylgalactosamine > α-Galactose	α-linked GalNAc structures	Dobbs et al., 1985
42	NPA_Narcissus pseudonarcissus	Mannose	PolyMannose structures with N-linkages	Kaku et al., 1990
43	PHA-E_Phaseolus vulgaris	Galactose	Gal(β -1,3(4)) GalNAc(β -1) linked complex structures	(*)
44	PHA-L_Phaseolus vulgaris	Galactose	Gal(β -1,3(4)) GalNAc(β -1) linked complex structures	(*)
45	PMA_Polygonatum multiflorum	Mannose	Not determined	Damme et al., 1996
46	PNA_Arachis hypogaea	β-Galactose	Gal(β 1-3)GalNac, Gal(β 1-4)GlcNAc and terminal β-Dgalactosyl residues	Lotan et al., 1975; Chandrasekaran et al., 2016
47	PSA_Pisum sativum	D-mannose>D-glucose	Branched mannose with α-1,6-linked fucose as determinant	Kornfeld et al., 1981; Debray and Montreuil, 1983
48	PSL_Polyporus squamosus	N-acetylneuraminic acid, Sialic Acid	Neu5Ac (α2-6) Gal	Zhang et al., 2001
49	PTA_Psophocarpus tetragonolobus	Galactose, N-acetylgalactosamine	GalNAc (α- 1,3) GalNAc, GalNAc (α-1,3) Gal	(*)
50	PWA_Phytolacca americana	N-acetylglucosamine	GlcNAc (b1,4) GlcNAc oligomers and [Gal-(b1,4) GlcNAc]2	(*)
51	RCA-I_Ricinus communis	β-Galactose, Lactose	Gal (β -1,4) GlcNAc β-1- linked	Lin and Li, 1980 ; Bhattacharyya et al., 1988; Chandrasekaran et al., 2016

52	RPA_Robinia pseudoacacia	N-Acetyl-D-galactosamine	Complex specificity	Wantyghem et al., 1986
53	SBA_Glycine max	α and β N-Acetyl-D-galactosamine>Galactose	GalNAc(β 1-3)Gal -R, GalNAc(β 1-4)Gal	Swamy et al., 1986; Bhattacharyya et al., 1988; Piller et al., 1990
54	SJA_Sophora japonica	N-Acetylgalactosamine and Galactose	Gal(β 1-3(4))GalNac	Poretz et al, 1974
55	SNA_Sambucus nigra	N-Acetyl-D-galactosamine>Galactose	GalNAc(β 1-6)Gal, GalNAc(α 1-6)Gal,GalNAc(α 1-3)Gal, Gal(β 1-6)Gal(β1-1)glycerol	Shibuya et al., 1987
56	SSA_Salvia sclare	α-N-Acetylgalactosamine	αGalNAc-Ser/Thr, GalNAc (β -1,3) GalNAc-R, GalNAc(β 1-3)Gal -R	Piller et al., 1990
57	STA_ Solanum tuberosum	β-N-Acetylgalactosamine	GlcNAc (b1,4) GlcNAc oligomers	Kawashima et al., 1990
58	TKA_Trichosanthes kirilowii	Galactose	Gal (β 1-4)GlcNAc internal clusters; polyvalent<br>Gal (β -1,4) GlcNAc β-1 linked; cluster forms of Gal (β -1,3) GlcNAc	Wu et al., 2000
59	TL_Tulipa sp.	N-acetylgalactosamine	GalNAc, Gal and Fuc residues of glycoconjugates	Cammue et al., 1986
60	UDA_Urtica dioica	N-Acetylglucosamine	GlcNAc oligomers	Shibuya et al., 1986
61	UEA-I_Ulex europaeus	Fucose, Arabinose	α1,2-linked fucose residues, Fuc (α 1-2)Gal (β1-4) GlcNAc	Allen et al., 1977; Gürtler, 1978
62	VFA_Vicia faba	Mannose > Glucose > N-Acetylglucosamine	Branched mannose with α-1,6-linked fucose as determinant	Debray, H., Montreuil, J., 1983
63	VGA_Vicia graminea	Galactose	Gal(β 1-3)GalNac (α 1-) linked	(*)

64	VRA_Vigna radiata	α-Galactose, Xylose, Methyl-α-D-galactopyranoside	Glycoconjugates containing terminal Gal residues	Suseelan et al., 1997
65	VVA_Vicia villosa	N-Acetylgalactosamine, Methyl-α-D-galactopyranoside	Gal(α 1-3)GalNac, GalNAc (α 1-)Ser/Thr	Tollefsen and Kornfeld, 1984; Piller et al., 1990
66	WFA_Wisteria floribunda	α and β N-Acetylgalactosamine > Galactose	αGalNAc(β 1-3)GalNAc, αGalNAc (β 1-3) Gal, βGalNAc (β 1-3(4)) Gal	Kurokawa et al., 1976; Piller et al., 1990
67	WGA_Triticum vulgaris	N-Acetylglucosamine	three β1-4 linked GluNAc residues, terminal β1-4 linked GalNAc, internal GlcNAc in the context of the Galβ1- 4GlcNAc, GalNAc β1-4 GlcNAc	Chandrasekaran et al., 2016
		GluNAc and GalNAc	GalNAcK1CSer/Thr (Tn), GalNAc at the nonreducing terminal, GlcNAcL1C at the non-reducing end and/or as an internal residue	Wu et al., 1998
Nr.	Alexa488 conjugated lectins	Single-sugar binding specificity	Carbohydrates linkage specificity	References (*)
1	AAL_Aleuria aurantia lectin	α-Fucose	Fuc(α -1,6)GlcNAc or Fuc(α -1,3) GalNAc related structures	Kochibe and Furukawa, 1980
2	CCA_Cancer antennarius	Sialic acid	O-linked sialic acid	(*)
3	Co_Codium fragile	N-acetylgalactosamine	GalNAc α1→ sequence	Wu et al., 1997
4	IAA_Iberis amara	N-acetyl-D-galactosamine	Complex specificity	(*)
5	MIA_Mangifera indica	N-acetyl-D-galactosamine	Not determined	(*)
6	PAA_Persea americana	Not inhibited	Not determined	(*)
7	PA-I_Pseudomonas aeruginosa	Galactose	Galα1→4Gal determinants	Gilboa-Garber, N., 1982

8	PPA_Ptilota plumosa	Galactose	terminal α-D-galactosyl residues	(*)
9	RTA_Trifolia repens	2-deoxy-glucose	not determined	(*)
10	SHA_Salvia horminum	N-acetylgalactosamine	not determined	Bird and Wingham, 1974
11	SSC_Sarothamnus scoparius (SAS-F)	α-Fucose	not determined	Gürtler, 1978

(*) ***For more information on the complex carbohydrates that most of these lectins can bind, please consult:***

Liener I. et al. (ed). (2012) - The lectins: properties, functions, and applications in biology and medicine. Elsevier.

Slifkin M. and Doyle RJ. (1990) - Lectins and their application to clinical microbiology. Clinical microbiology reviews, 3(3), pp.197-21

Van Damme EJM, et al. (1998) - Handbook of plant lectins: properties and biomedical applications. John Wiley & Sons.

Wu AM. (2001) - The Molecular Immunology of Complex Carbohydrates-2, Springer US.

MyBioSource webpage - https://www.mybiosource.com/

Vector Laboratories webpage - https://vectorlabs.com/

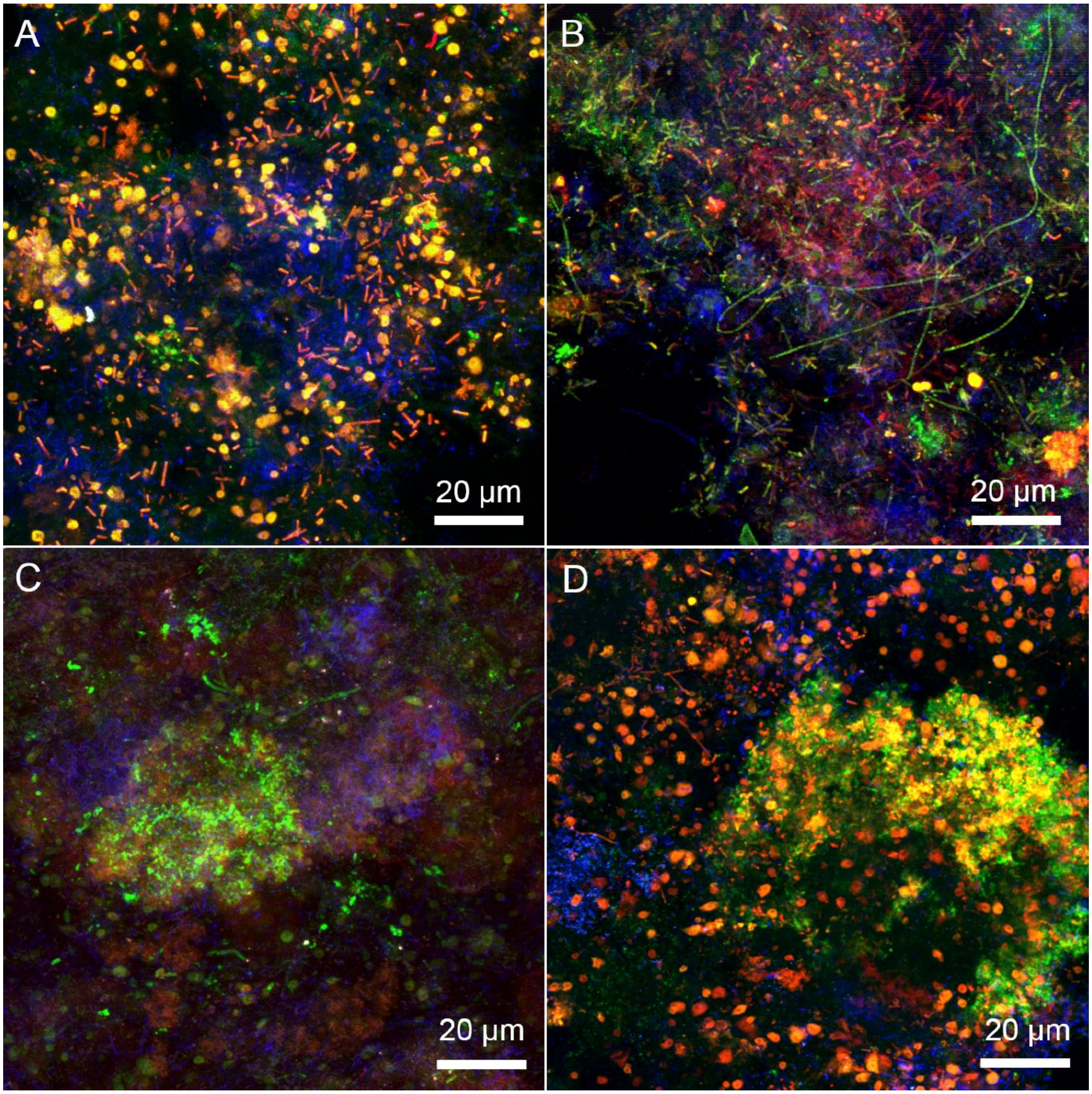

Fig.S5 – CLSM maximum intensity projections of lectin combinations on LS granules showing different glycoconjugate patterns as well as co-localization. In (A), PMA in green and WGA in red; in (B), HAA in green and WGA in red; in (C), HAA in green and RPA in red; in (D), HAA in green and AAL in red. Blue is the F_{420} autofluorescence from methanogenic cells. Yellow indicates co-localisation of the two lectin signals.

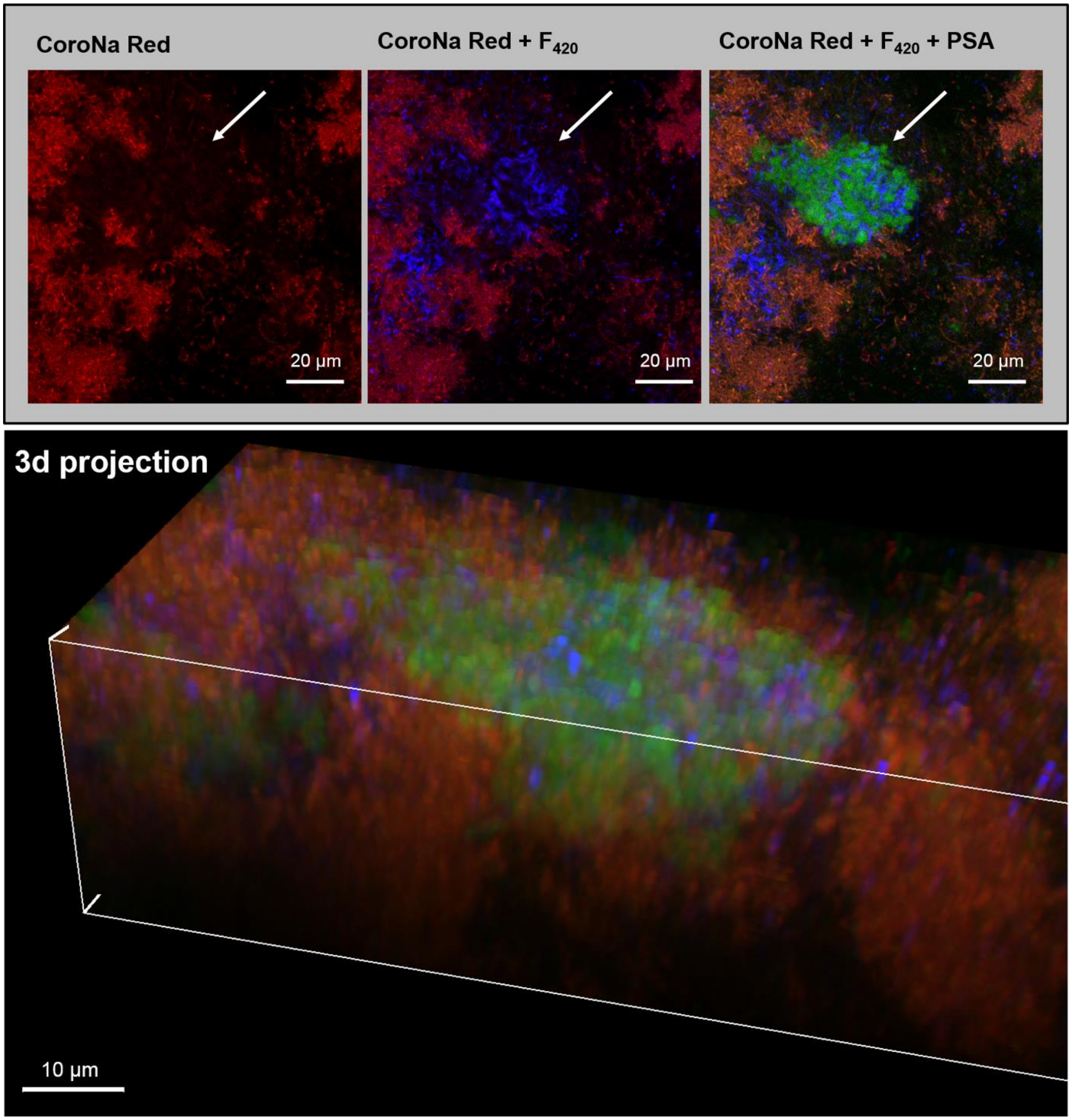

Fig. S6 – CLSM fluorescent images and 3D projection of the HS reactor (20 g/L Na^+) granules analysed by combining PSA lectin (in green) with CoroNa Red staining. In blue F_{420} autofluorescent methanogens. Top: Images showing the same location by adding individual channels. Bottom: 3d projection of the same dataset. White arrows indicate the spots occupied by the cloudy EPS structure that were not positive to CoroNa Red.

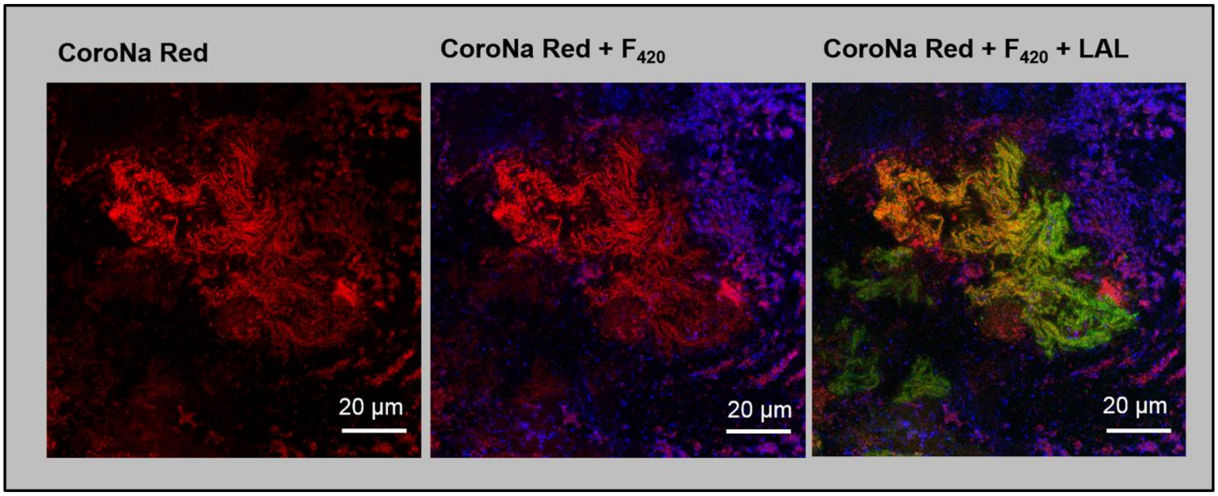

Fig. S7 – CLSM fluorescent images of the HS reactor (20 g/L Na^+) granules analysed by combining LAL lectin (in green) with CoroNa Red staining. In blue F_{420} autofluorescent methanogens. Images are showing the same location by adding individual channels.

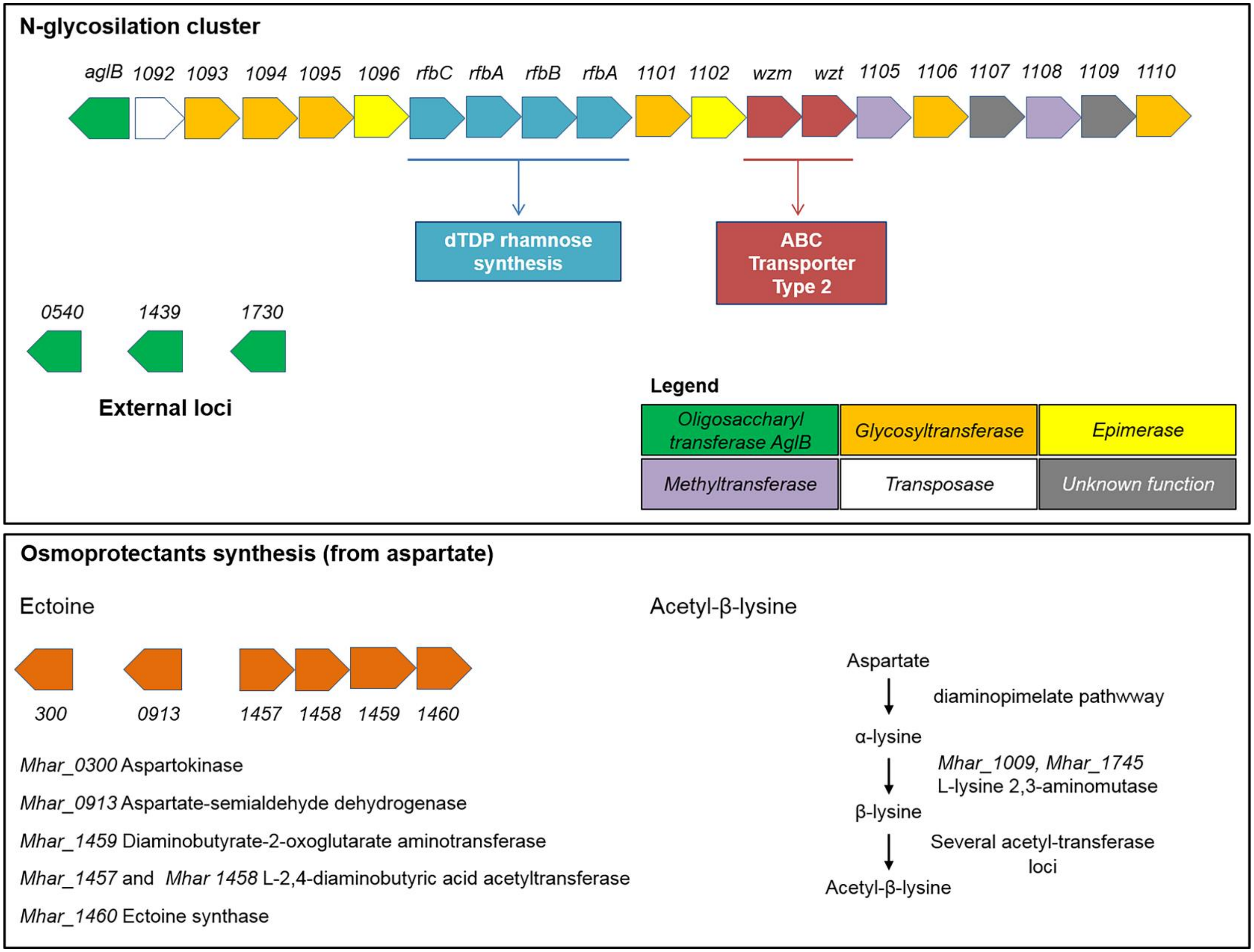

Fig.S8 – Schematic representation showing the presence in the *M.harundinacea* 6Ac genome of a cluster for the N-glycosilation pathway (loci 1091 to 1110) and of the genes encoding for the ectoine and acetyl-β-lysine synthesis. The clustering of putative N-glycosilation genes in *M.harundinacea* is a unique feature within the available genomic data in the international databases within to the genus *Methanosaeta*.

- **Reconstruction of *M.harundinacea* 6Ac genetic loci**

To define the salinity stress genetic features of *M.harundinacea*, the complete genome of *M.harundinacea* strain 6Ac (Zhu et al. 2012) was used as reference. On the basis of literature data on salinity stress related genes (Peters et al., 1990; Rocchetta and Lam, 1997; Pflüger, 2003; Guan, 2012; Jarrell, 2014), the search and analysis of the annotated loci within the strain 6Ac genome, their genomic context and the conserved regions were carried out through the NCBI Genome database tools (https://www.ncbi.nlm.nih.gov/genome/).

The corresponding proteins functions and domains were checked with UniProt (http://www.uniprot.org/), while the connected microbial pathways were investigated via the KEGG pathway maps (http://www.genome.jp/kegg-bin/get_htext?br08901).

- **References**

Allen, A.K., 1995. Purification and characterization of an N-acetyllactosamine-specific lectin from tubers of Arum maculatum. BBA - Gen. Subj. 1244, 129–132.

Allen, H.J., Johnson, E.A. and Matta, K.L., 1977. A comparison of the binding specificities of lectins from Ulex europaeus and Lotus tetragonolobus. Immunological communications, 6(6), pp.585-602.

Amann, R., Binder, B., 1990. Combination of 16S rRNA-targeted oligonucleotide probes with flow cytometry for analyzing mixed microbial populations. Appl. enviromental Microbiol. 56, 1919–25.

Battison, A.L., Summerfield, R.L., 2009. Isolation and partial characterisation of four novel plasma lectins from the American lobster Homarus americanus. Dev. Comp. Immunol. 33, 198–204.

Berman, E., Brown, J.H., Lis, H., Sharon, N., 1985. Binding of [1-13C] galactose-labeled N-acetyllactosamine to Erythrina cristagalli agglutinin as studied by 13C-NMR. Eur. J. Biochem. 152.

Bhattacharyya, L., Haraldsson, M., Brewer, C.F., 1988. Precipitation of galactose-specific lectins by complex-type oligosaccharides and glycopeptides: studies with lectins from Ricinus communis (agglutinin I), Erythrina indica, Erythrina arborescens, Abrus precatorius (agglutinin), and Glycine max (soybean). Biochemistry 27, 1034–41.

Bird, G.W.G. and Wingham, J., 1974. Haemagglutinins from salvia. Vox sanguinis, 26(2), pp.163-166.

Björnsson, L., Hugenholtz, P., Tyson, G.W., Blackall, L.L., 2002. Filamentous Chloroflexi (green non-sulfur bacteria) are abundant in wastewater treatment processes with biological nutrient removal. Microbiology 148, 2309–2318.

Bloch, R., Jenkins, J., Roth, J., Burger, M.M., 1976. Purification and characterization of two lectins from Caragana arborescens seeds. J. Biol. Cheminstry 251, 5929–5935.

Cammue, B.P.A., Peeters, B. and Peumans, W.J., 1986. A new lectin from tulip (Tulipa) bulbs. Planta, 169(4), pp.583-588.

Chandrasekaran, E.V., Xue, J., Xia, J., Khaja, S.D., Piskorz, C.F., Locke, R.D., Neelamegham, S., Matta, K.L., 2016. Novel interactions of complex carbohydrates with PNA, RCA-I, SNA-I and WGA agglutinins as revealed by the binding specificities of these lectins towards mucin core-2 O-linked and N-linked glycans and related structures. Glycoconj J 33, 819.

Crocetti, G., Murto, M., Björnsson, L., 2006. An update and optimisation of oligonucleotide probes targeting methanogenic Archaea for use in fluorescence in situ hybridisation (FISH). J. Microbiol. Methods 65, 194–201.

Dam, T.K., Bachhawat, K., Rani, P.G., Surolia, A., 1998. Garlic (Allium sativum) lectins bind to high mannose oligosaccharide chains. J. Biol. Chem. 273, 5528–5535.

Damme, E.J., Barre, A., Rougé, P., Leuven, F., Balzarini, J. and Peumans, W.J., 1996. Molecular cloning of the lectin and a lectin-related protein from common Solomon's seal (Polygonatum multiflorum). Plant molecular biology, 31(3), pp.657-672.

Debray, H., Montreuil, J., 1983. Structural basis for the affinity of four insolubilized lectins, with a specificity for α-D-mannose, towards various glycopeptides with the N-glycosylamine linkage and related oligosaccharides. J. Biosci. 5, 93–100.

Dobbs, L.G., Williams, M.C., Brandt, A.E., 1985. Changes in biochemical characteristics and pattern of lectin binding of alveolar type II cells with time in culture. Biochim. Biophys. Acta 846, 155–166.

Fountain, D.W., Campbell, B.A., 1984. A lectin isolate from mucus of Helix aspersa. Comp. Biochem. Physiol. -- Part B Biochem. 77, 419–425.

Gagliano, M.C., Braguglia, C.M., Gianico, a., Mininni, G., Nakamura, K., Rossetti, S., 2015. Thermophilic anaerobic digestion of thermal pretreated sludge: Role of microbial community structure and correlation with process performances. Water Res. 68, 498–509.

Geisler, C. and Jarvis, D.L., 2011. Letter to the Glyco-Forum: Effective glycoanalysis with Maackia amurensis lectins requires a clear understanding of their binding specificities. Glycobiology, 21(8), pp.988-993.

Gercken, J., Renwrantz, L., 1994. A new mannan-binding lectin from the serum of the eel (Anguilla anguilla L.): isolation, characterization and comparison with the fucose-specific serum lectin. Comp. Biochem. Physiol. -- Part B Biochem. 108, 449–461.

Gilboa-Garber, N., 1982. Pseudomonas aeruginosa lectins. Methods in enzymology, 83, pp.378-385.

Goldstein, I.J., Kobata, A., 1987. Carbohydrate binding properties of complex-type oligosaccharides on immobilized Datura stramonium lectin. J. Biol. Chem. 262, 1602–1607.

Guan, Z., Naparstek, S., Calo, D., and Eichler, J. 2012. Protein glycosylation as an adaptive response in Archaea: growth at different salt concentrations leads to alterations in Haloferax volcanii S-layer glycoprotein N-glycosylation. 14, 743–753.

Gupta, D., Oscarson, S., Raju, T.S., Stanley, P., Toone, E.J. and Brewer, C.F., 1996. A comparison of the fine saccharide-binding specificity of Dioclea grandiflora lectin and concanavalin A. European journal of biochemistry, 242(2), pp.320-326.

Gürtler, L.G., 1978. The fucosyl specific lectins of Ulex europaeus and Sarothamnus scoparius biochemical characteristics and binding properties to human B-lymphocytes. BBA - Gen. Subj. 544, 593–604.

Hardman, K.D. and Ainsworth, C.F., 1976. Structure of the concanavalin A-methyl α-D-mannopyranoside complex at 6-Å resolution. Biochemistry, 15(5), pp.1120-1128.

Jarrell, K. F., Ding, Y., Meyer, B. H., Albers, S.-V., Kaminski, L., and Eichler, J. 2014. N-Linked Glycosylation in Archaea: a Structural, Functional, and Genetic Analysis. Microbiol. Mol. Biol. Rev. 78, 304–341.

Kaku, H., Van Damme, E.J.M., Peumans, W.J., Goldstein, I.J., 1990. Carbohydrate-binding specificity of the daffodil (Narcissus pseudonarcissus) and amaryllis (Hippeastrum hybr.) bulb lectins. Arch. Biochem. Biophys. 279, 298–304.

Kawashima, H., Sueyoshi, S., Li, H., Yamamoto, K., Osawa, T., 1990. Carbohydrate binding specificities of several poly-N-acetyllactosamine-binding lectins. Glycoconj. J. 7, 323–334.

Kirkeby, S., Winter, H.C., Goldstein, I.J., 2004. Comparison of the binding properties of the mushroom Marasmius oreades lectin and Griffonia simplicifolia I-B4 isolectin to αgalactosyl carbohydrate antigens in the surface phase. Xenotransplantation 11, 254–261.

Knibbsso, R.N., Scott, E., Glickll, G.D., Goldstein, I.J., 1993. Binding Determinants of the Sialic Acid-specific Lectin from the Slug Limax flavus. J. Biol. cheminstry 268, 18524–18531.

Kochibe, N., Furukawa, K., 1980. Purification and properties of a novel fucose-specific hemagglutinin of Aleuria aurantia. Biochemistry 19, 2841–2846.

Kong, Y., He, M., McAlister, T., Seviour, R., Forster, R., 2010. Quantitative fluorescence in situ hybridization of microbial communities in the rumens of cattle fed different diets. Appl. Environ. Microbiol. 76, 6933–6938.

Kornfeld, K., Reitman, M.L., Kornfeld, R., 1981. The carbohydrate-binding specificity of pea and lentil lectins. Fucose is an important determinant. J. Biol. Chem. 256, 6633–6640.

Kurokawa, T., Tsuda, M., Sugino, Y., 1976. Purification and Characterization Seeds of a Lectin from Wistaria floribunda seeds. J. Biol. Chem. 251, 5686–5693.

Lin, T.T.S., Li, S.S.-L., 1980. Purification and Physicochemical Properties of Ricins and Agglutinins from Ricinus communis. Eur. J. Biochem. 105, 453–459.

Liu, J.R. and Seviour, R.J., 2001. Design and application of oligonucleotide probes for fluorescent in situ identification of the filamentous bacterial morphotype Nostocoida limicola in activated sludge. Environmental microbiology, 3(9), pp.551-560

Lotan, R., Skutelsky, E., Danon, D., Sharon, N., 1975. The purification, composition, and specificity of the anti-T lectin from peanut (Arachis hypogaea). J. Biol. Chem. 250, 8518–8523.

Lücker, S., Steger, D., Kjeldsen, K.U., MacGregor, B.J., Wagner, M., Loy, A. (2007) Improved 16S rRNA-targeted probe set for analysis of sulfate-reducing bacteria by fluorescence in situ hybridization. J. Microbiol. Methods 69, 523–528.

Manz, W., Amann, R., Ludwig, W., Wagner, M., Schleifer, K.H. (1992) Phylogenetic oligodeoxynucleotide probes for the major subclasses of Proteobacteria – problems and solutions. Syst. Appl. Microbiol. 15, 593–600.

Meier, H., Amann, R., Ludwig, W., Schleifer, K.H., 1999. Specific oligonucleotide probes for in situ detection of a major group of gram-positive bacteria with low DNA G + C content. Syst. Appl. Microbiol. 22, 186–96.

Nakamura-Tsuruta, S., Kominami, J., Kuno, A., Hirabayashi, J., 2006. Evidence that Agaricus bisporus agglutinin (ABA) has dual sugar-binding specificity. Biochem. Biophys. Res. Commun. 347, 215–220.

Nakamura-Tsuruta, S., Uchiyama, N., Peumans, W.J., Van Damme, E.J.M., Totani, K., Ito, Y., Hirabayashi, J., 2008. Analysis of the sugar-binding specificity of mannose-binding-type Jacalin-related lectins by frontal affinity chromatography - An approach to functional classification. FEBS J. 275, 1227–1239.

Neef, a, Amann, R., Schlesner, H., Schleifer, K.H., 1998. Monitoring a widespread bacterial group: in situ detection of planctomycetes with 16S rRNA-targeted probes. Microbiology 144 (3257–66).

Pereira, M.E., Kabat, E. a, 1974. Specificity of purified hemagglutinin (lectin) from Lotus tetragonolobus. Biochemistry 13, 3184–3192.

Peters, P., Galinski, E. A., and Trüper, H. G. 1990. The biosynthesis of ectoine. FEMS Microbiol. Lett. 71, 157–162.

Peumans, W.J., Nsimba-Lubaki, M., Carlier, A.R. and Driessche, E., 1984. A lectin from Bryonia dioica root stocks. Planta, 160(3), pp.222-228.

Pflüger, K., Baumann, S., Gottschalk, G., Lin, W., Santos, H., and Müller, V., 2003. Lysine-2,3-Aminomutase and β-Lysine Acetyltransferase Genes of Methanogenic Archaea Are Salt Induced and Are Essential for the Biosynthesis of Nε-Acetyl-β-Lysine and Growth at High Salinity. Appl. Environ. Microbiol. 69, 6047–6055. doi:10.1128/AEM.69.10.6047-6055.2003.

Piller, V., Piller, F., Cartron, J. -P, 1990. Comparison of the carbohydrate-binding specificities of seven N-acetyl-D-galactosamine-recognizing lectins. Eur. J. Biochem. 191, 461–466.

Piskarev, V.E., Bushueva, T.L., Iamskov, I.A., 2007. Interaction of the Laburnum anagyroides lectin with fucoantigens. Russ. J. Bioorganic Chem. 33, 170–174.

Poretz, R.D., Riss, H., Timberlake, J.W. and Chien, S.M., 1974. Purification and properties of the hemagglutinin from Sophora japonica seeds. Biochemistry, 13(2), pp.250-256.

Presant, C.A. and Kornfeld, S., 1972. Characterization of the cell surface receptor for the Agaricus bisporus hemagglutinin. Journal of Biological Chemistry, 247(21), pp.6937-6945.

Raskin, L., Stromley, J.M., Rittmann, B.E., Stahl, D. a, 1994. Group-specific 16S rRNA hybridization probes to describe natural communities of methanogens. Appl. Environ. Microbiol. 60, 1232–40.

Rinderle, S.J., Goldstein, I.J., Matta, K.L., Ratcliffe, R.M., 1989. Isolation and characterization of amaranthin, a lectin present in the seeds of Amaranthus caudatus, that recognizes the T- (or cryptic T)-antigen. J. Biol. Chem. 264, 16123–16131.

Rocchetta, H. L., and Lam, J. S., 1997. Identification and functional characterization of an ABC transport system involved in polysaccharide export of A-band lipopolysaccharide in Pseudomonas aeruginosa. J. Bacteriol. 179, 4713–4724.

Sastry, M.V.K., Surolia, A., 1986. Intrinsic fluorescence studies on saccharide binding to Artocarpus integrifolia lectin. Biosci. Rep. 6, 853–860.

Schlegel, K., Müller, V., 2013. Evolution of Na^+ and H^+ bioenergetics in methanogenic archaea. Biochem. Soc. Trans. 41, 421–426.

Shibata, S., Goldstein, I.J., Baker, D.A., 1982. Isolation and characterization of a Lewis b-active lectin from Griffonia simplicifolia seeds. J. Biol. Chem. 257, 9324–9329.

Shibuya, N., Goldstein, I.J., Broekaert, W.F., Nsimba-Lubaki, M., Peeters, B., Peumans, W.J., 1987. The elderberry (Sambucus nigra L.) bark lectin recognizes the Neu5Ac(alpha 2-6)Gal/GalNAc sequence. J. Biol. Chem. 262, 1596–1601.

Shibuya, N., Goldstein, I.J., Shafer, J.A., Peumans, W.J., Broekaert, W.F., 1986. Carbohydrate binding properties of the stinging nettle (Urtica dioica) rhizome lectin. Arch. Biochem. Biophys. 249, 215–224.

Shibuya, N., Goldstein, I.J., Van Damme, E.J., Peumans, W.J., 1988. Binding properties of a mannose-specific lectin from the snowdrop (Galanthus nivalis) bulb. J. Biol. Chem. 263, 728–734.

Singh, D.D., Saikrishnan, K., Kumar, P., Surolia, A., Sekar, K., Vijayan, M., 2005. Unusual sugar specificity of banana lectin from Musa paradisiaca and its probable evolutionary origin. Crystallographic and modelling studies. Glycobiology 15, 1025–1032.

Stahl, D.A., Amann, R. 1991 Development and application of nucleic acid probes. In: Stackebrandt, E., Goodfellow, M. (Eds.), Nucleic acid techniques in bacterial systematics, John Wiley & Sons, Chichester, pp. 205–248.

Sudmalis, D., Gagliano, M. C., Pei, R., Grolle, K., Plugge, C. M., Rijnaarts, H. H. M., et al. (2017). Fast anaerobic sludge granulation at elevated salinity. Water Res. 128, 293–303.

Suseelan, K.N., Bhatia, C.R., Mitra, R., 1997. Characteristics of two major lectins from mungbean (Vigna radiata) seeds. Plant Foods Hum. Nutr. 50, 211–222.

Swamy, M.J., Sastry, M.V.K., Khan, M.I., Surolia, A., 1986. Thermodynamic and kinetic studies on saccharide binding to soya-bean agglutinin. Biochem. J.

Tollefsen, S.E., Kornfeld, R., 1984. The B4 lectin from Vicia villosa seeds interacts with N-acetylgalactosamine residues on erythrocytes with blood group Cad specificity. Biochem. Biophys. Res. Commun. 123, 1099–1106.

Trebesius, K., Leitritz, L., Adler, K., Schubert, S., Autenrieth, I.B., Heesemann, J., 2000. Culture independent and rapid identification of bacterial pathogens in necrotising fasciitis and streptococcal toxic shock syndrome by fluorescence in situ hybridisation. Med. Microbiol. Immunol. 188, 169–175.

Wang, W., Peumans, W.J., Rougé, P., Rossi, C., Proost, P., Chen, J., Van Damme, E.J.M., 2003. Leaves of the Lamiaceae species Glechoma hederacea (ground ivy) contain a lectin that is structurally and evolutionary related to the legume lectins. Plant J. 33, 293–304.

Wantyghem, J., Goulut, C., Frénoy, J.P., Turpin, E., Goussault, Y., 1986. Purification and characterization of Robinia pseudoacacia seed lectins. A re-investigation. Biochem. J. 237, 483–9.

Wu, A.M., Song, S.C., Chang, S.C., Wu, J.H., Chang, K.S.S., Kabat, E.A., 1997. Further characterization of the binding properties of a GalNAc specific lectin from Codium fragile subspecies tomentosoides. Glycobiology 7, 1061–1066.

Wu, A.M., Wu, J.H., Chen, Y.Y., Tsai, M.S., Herp, A., 1999. Forssman pentasaccharide and polyvalent Galβ1→4GlcNAc as major ligands with affinity for Caragana arborescens agglutinin. FEBS Lett. 463, 225–230.

Wu, A.M., Wu, J.H., Tsai, M.S., Herp, A., 2000. Carbohydrate specificity of an agglutinin isolated from the root of Trichosanthes kirilowii. Life Sci 66, 2571–2581.

Yamamoto, K., Konami, Y., Osawa, T., 1992. Determination of the carbohydrate-binding site of Bauhinia purpurea lectin by affinity chromatography. J. Chromatogr. A 597, 221–230.

Yamashita, K., Totani, K., Ohkura, T., Takasaki, S., Goldstein, I.J., Kobata, A., 1987. Carbohydrate binding properties of complex-type oligosaccharides on immobilized Datura stramonium lectin. J. Biol. Chem. 262, 1602–1607.

Zhang, B., Palcic, M.M., Mo, H., Goldstein, I.J. and Hindsgaul, O., 2001. Rapid determination of the binding affinity and specificity of the mushroom Polyporus squamosus lectin using frontal affinity chromatography coupled to electrospray mass spectrometry. Glycobiology, 11(2), pp.141-147.
